# Cryo-EM structures of a LptDE transporter in complex with Pro-macrobodies offer insight into lipopolysaccharide translocation

**DOI:** 10.1038/s41467-022-29459-2

**Published:** 2022-04-05

**Authors:** Mathieu Botte, Dongchun Ni, Stephan Schenck, Iwan Zimmermann, Mohamed Chami, Nicolas Bocquet, Pascal Egloff, Denis Bucher, Matilde Trabuco, Robert K. Y. Cheng, Janine D. Brunner, Markus A. Seeger, Henning Stahlberg, Michael Hennig

**Affiliations:** 1 leadXpro AG, Park Innovaare, 5234 Villigen, Switzerland; 2 C-CINA, Biozentrum, University of Basel, Mattenstr. 24, 4058 Basel, Switzerland; 3 Institute of Medical Microbiology, University of Zürich, Gloriastasse 28/30, 8006 Zürich, Switzerland; 4 Laboratory of Biomolecular Research, Division of Biology and Chemistry, Paul Scherrer Institute (PSI), 5232 Villigen, Switzerland; 5 VIB-VUB Center for Structural Biology, VIB, Belgium; Structural Biology Brussels, Vrije Universiteit Brussel, 1050 Brussels, Belgium; 6 Present Address: VIB-VUB Center for Structural Biology, VIB, 1050 Brussels, Belgium; 7 Present Address: Linkster Therapeutics AG, 8006 Zürich, Switzerland

**Keywords:** Cryoelectron microscopy, Protein design, Synthetic biology, Bacterial structural biology, Membrane proteins

## Abstract

Lipopolysaccharides are major constituents of the extracellular leaflet in the bacterial outer membrane and form an effective physical barrier for environmental threats and for antibiotics in Gram-negative bacteria. The last step of LPS insertion via the Lpt pathway is mediated by the LptD/E protein complex. Detailed insights into the architecture of LptDE transporter complexes have been derived from X-ray crystallography. However, no structure of a laterally open LptD transporter, a transient state that occurs during LPS release, is available to date. Here, we report a cryo-EM structure of a partially opened LptDE transporter in complex with rigid chaperones derived from nanobodies, at 3.4 Å resolution. In addition, a subset of particles allows to model a structure of a laterally fully opened LptDE complex. Our work offers insights into the mechanism of LPS insertion, provides a structural framework for the development of antibiotics targeting LptD and describes a highly rigid chaperone scaffold to enable structural biology of challenging protein targets.

## Abstract (web summary)

Lateral opening of the LptDE transporter in the outer membrane of Gram-negative bacteria is necessary for insertion of lipopolysaccharides. Here, Botte et al. report a cryo-EM structure of a partially opened LptDE transporter, in complex with rigid chaperones derived from nanobodies.

## Introduction

Multi-drug-resistant bacteria present a growing concern for human health^1^. Among these, Gram-negative bacteria are especially problematic, because they are well-shielded from their environment by an outer membrane (OM) that establishes a tight barrier for several antibiotics due to the high density of lipopolysaccharides (LPS) in the outer leaflet of the membrane^2^. LPS are thus an essential component of the bacterial resistance. LPS is synthesized in the cytosol and transported across the periplasmic space to the outer leaflet via the Lpt pathway, consisting of the proteins LptA, B, C, D, E, F, and G, which form a trans-envelope complex to bridge the inner and outer membranes^3–6^. Hence, disrupting the assembly of the outer membrane by inhibiting the Lpt pathway is an attractive strategy for novel antibiotic therapeutics^7,8^. The membrane-integral LptDE complex in the OM executes the last step of LPS transport^9^ and is among other OM-protein constituents a promising antibiotic target also due to its surface-exposed localization^10,11^. The structures of LptDE from multiple species were determined by X-ray crystallography and provided detailed insight into the general architecture and the LPS path^12–14^. The lateral opening of the LptD β-barrel^15^, which enables the exit of LPS into the outer leaflet, has been conclusively inferred from simulations and mutagenesis studies^16,17^, yet awaits structural evidence. From a pharmaceutical perspective, open conformations of barrel architectures are of high interest because of the propensity of β-hairpin mimetics (currently the most promising class of antibiotics to interfere with OM-assembly proteins^10,11,18^) to target to terminal β-strands by β-augmentation^19^. Thus, more insight into the conformational space of LptD is in demand not only for an understanding of LPS transport in general but also for structure-based drug design. Here, we present cryo-EM structures of the LptDE transporter of the pathogen *Neisseria gonorrhoeae* (NgLptDE) with partially and fully opened lateral gates. Importantly, we could obtain these structures by complexation with nanobody-based chaperones. Departing from the structure of the previously described macrobodies^20^, chaperones that are built of a nanobody (Nb), and a C-terminally fused maltose-binding protein (MBP), we increased the rigidity of the original linker between the two moieties substantially to design an improved scaffold, named Pro-Macrobodies (PMbs), with excellent properties for particle classification and particle enlargement in cryo-EM.

## Results

### Design of pro-macrobodies and complexation with NgLptDE for cryo-EM

**Fig. 1:**
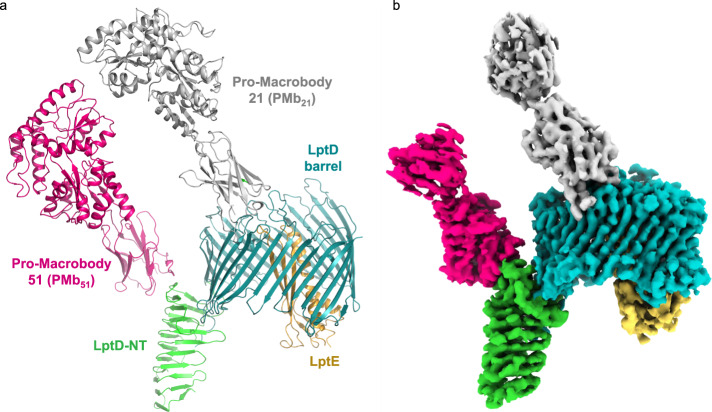
Cryo-EM structure of the NgLptDE–PMb_21_/PMb_51_ complex. **a** Side view of the NgLptDE–PMb_21_/PMb_51_ complex. **b** Cryo-EM map of the NgLptDE–PMb_21_/PMb_51_ complex at 3.4 Å. Colors encode domains as in (**a**).

**Fig. 2:**
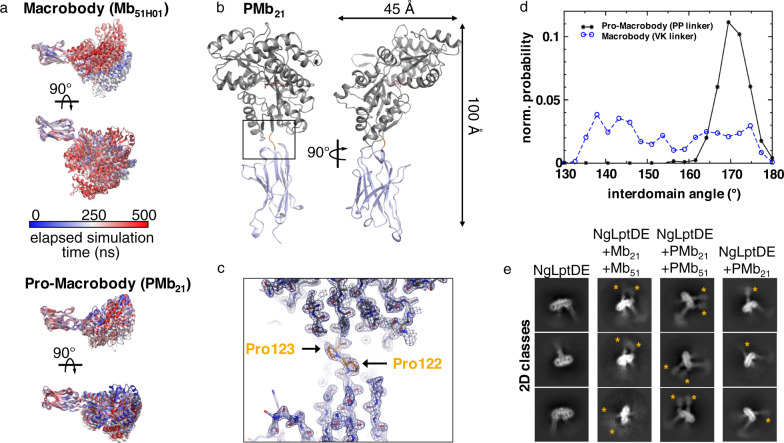
Generation and properties of pro-macrobodies. **a** Conformational flexibility between the Nb- and MBP moieties connected by a Val–Lys linker (top) and Pro–Pro linker (bottom). Mb_51H01_ from PDB entry 6HD8 was subjected to a 500 ns all-atom MD simulation. Frames are colored by elapsed simulation time (middle panel) and aligned on the Nb moiety to display the relative movements of MBP. The simulation was repeated with PMb_21_ using the X-ray structure shown in (**b**). **c** Enlarged view (boxed in (**b**)) of the linker in the PMb_21_ crystal structure with the map contoured at (**d**) Plot of the normalized probability versus the interdomain angle between the Nb and MBP in Mbs (Val–Lys linker) and PMbs (Pro–Pro linker), as derived from MD simulations. The PMbs (black asterisks, solid line) show a defined distribution peaking at 170 ± 5°, whereas the original linker of macrobodies shows a wide distribution spanning almost 50° (blue circles, dashed line). **e** 2D-class averages of NgLptDE uncomplexed (left), complexed with Mbs 21 and 51 (left middle), complexed with PMbs 21 and 51 (middle right), and complexed with PMb21 (right). The MBP moiety is indicated with yellow asterisks. Source data are provided as a Source Data file.

To structurally characterize LptDE of *N. gonorrhoeae* and open the possibility of finding new conformations of these transporters, we used single-particle cryo-electron microscopy (cryo-EM) as this method provides an opportunity to capture the conformational breadth of protein samples^21–23^. After extensive optimization, we obtained a high-resolution structure of NgLptDE at 3.4 Å in complex with enlarged variable domains of heavy-chain antibodies (VHHs)—PMb_21_ and PMb_51_ (Fig. 1a, b; Supplementary Figs. 1a–d, 4a–c). Our initial attempts to obtain a structure of uncomplexed NgLptDE by cryo-EM were limited to a resolution of 4.6 Å (Supplementary Figs. 1e–f). Consequently, molecular details such as side chains for confident tracing of the sequence during de novo building and the identification of potential ligands could not be visualized. During the course of our study, we have generated sybodies (Sbs), which are synthetic nanobodies (Nbs) or VHHs^24^ against NgLptDE (Supplementary Fig. 7a–c). Sbs are generated by ribosome display from libraries encoding for synthetic nanobodies having variation of the complementarity-determining region (CDR) loops. Sbs can be selected in regimes that are impossible to put into practice in an animal body, such as toxic conditions (citation 24 again?). In addition, this approach has also the advantage to be faster than the traditional immunization method. We thought to transform the obtained Sbs to macrobodies (Mbs) by fusion of MBP to the C-terminus, as recently described for the crystallization of an ion channel^20^ to use them as fiducial markers for improved particle classification. Since a chaperone for cryo-EM requires rigidity, the flexibility of Mb_51H01_ (extracted from the PDB entry 6HD8) (Fig. 2a) was initially assessed by molecular-dynamics (MD) analysis. A bending motion of the MBP moiety was observed relative to the Nb of ~50° in multiple directions as well as torsional movements (Fig. 2a, d) due to high rotational freedom of the linker residues Val122 and Lys123 of Mb_51H01_ (Supplementary Fig. 2c). Despite their promising shape and size, Mbs are thus of limited use for particle enlargement in cryo-EM. In silico design of more rigid linkers was attempted. In particular, substitutions of Val122 and Lys123 by two consecutive prolines was found to lead to a stable chaperone where motions are dampened as proline has the lowest rotational freedom of all amino acids. The mutated model was computationally predicted to adopt a new conformation, where the MBP moiety is turned by ~170 degrees (Supplementary Fig. 2b) according to the trans-configuration between Pro122 and Pro123 (Fig. 2c, Supplementary Fig. 2e). The mutated Mb, termed Pro-Macrobody (PMb), showed strongly reduced flexibility in MD simulations (Fig. 2a,d; Supplementary Fig. 2c) and retained the positive properties of Mbs such as their simple design and their elongated structure. We then transformed Sbs against NgLptDE into PMbs (Supplementary Fig. 2a) and crystallized PMb_21_. The X-ray structure of PMb_21_ at a resolution of 2 Å confirmed the predicted structure and showed the lowest B-factors around its linker region that forms a short poly-Pro helix-II stretch (Fig. 2b, c; Supplementary Fig. 2d, e; Supplementary Table 2). The C-terminal end of the Sb moiety and the N-terminal region of MBP feature rigid β-sheets that are only bridged by the intrinsically stiff di-proline linker without additional interactions that would stabilize interdomain motions (Supplementary Fig. 2e). The specific binding moiety could thus be exchanged without a change in the properties of the connection. Further, the PMbs retained their binding kinetics compared with the original Sbs using grating-coupled interferometry (GCI), a biophysical characterization method similar to surface-plasmon resonance (Supplementary Fig. 7c). Next, using only monomeric NgLptDE (Supplementary Fig. 3a, b; see below), we identified a complex of NgLptDE with PMb_21_ and PMb_51_ (Supplementary Fig. 3c–e), two of the strongest binders from our analysis, using size-exclusion chromatography (SEC) (Supplementary Fig. 7a, c). We subjected this quaternary complex to cryo-EM and obtained a map of high quality (Fig. 1b, Supplementary Table. 1, Supplementary Fig. 1c, d) that significantly improved over the uncomplexed NgLptDE (Supplementary Fig. 1g, h). The rigidity of the PMbs is apparent from 2D-class averages (Fig. 2e) where the N- and C-terminal lobes of MBP are clearly recognizable. The respective identities of PMb_21_ and PMb_51_ could be assigned by 2D-class averages of a NgLptDE–PMb_21_ complex (Fig. 2e). PMb_21_ and PMb_51_ appear very similar in 2D-class averages in support of the universality and rigidity of the PMb scaffold. When we subjected an NgLptDE complex with Mb_21_ and Mb_51_ (with the original Val–Lys linker) to cryo-EM, it was apparent from 2D-class averages that these chaperones are more flexible, and thus, the MBP moiety is much less defined (Fig. 2e).

### The cryo-EM structure of NgLpTDE reveals a partially open LptD barrel

**Fig. 3:**
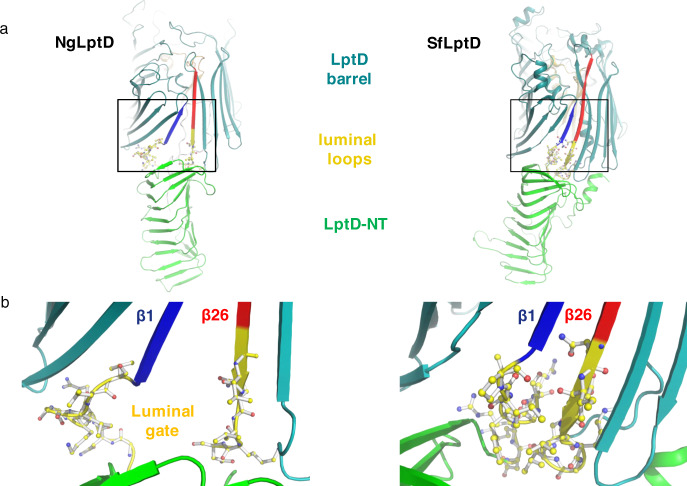
The luminal gate in structures of NgLptDE and SfLptDE. **a** The luminal turns (yellow) of NglptDE (left) are separated by more than 3 Å, leading to a continuous groove between the LptD barrel and the hydrophobic groove of the N-terminal domain. In SfLptDE (PDB-ID 4Q35) (right), the luminal turns obstruct the luminal gate. **b** Enlarged view of the luminal gates of NgLptDE (left) and SfLptDE (right). The first (β1) and last (β26) strands of the LptD barrels are shown in blue and red, respectively.

**Fig. 4:**
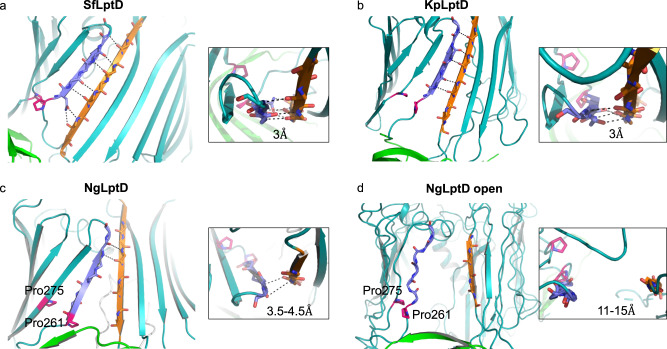
Hydrogen bonds between the β-strands 1 and 26 in structures of the LptD barrel. **a** SfLptDE, (**b**) KpLptDE (PDB-ID 5IV9), (**c**) NgLptDE (partially open), and (**d**) NgLptDE (open lateral gate). Distances between the terminal strands are indicated and small insets show a view from extracellular. β-strand 1 and β26 are shown in light blue and orange, respectively, and the conserved proline residues of β-strands 1 and 2 in pink.

The cryo-EM structure of the complex showed the same overall LptDE architecture observed also in X-ray structures from LptDE of other Gram-negative bacteria^12–14^ (Figs. 1a, 3a), but revealed also significant differences. Whereas NgLptE in our structure is nearly identical to LptE structures from other bacteria, our structure of NgLptD shows important deviations. The lateral and luminal gates in the cryo-EM structure of NgLptDE are more open compared with LptDE of *Klebsiella pneumoniae* (KpLptDE) and even more so compared with the X-ray structure of *Shigella flexneri* LptDE (SfLptDE)^12–14^ (Fig. 4a–c). Only three hydrogen bonds were observed between β-sheets β1 and β26, which increases the separation of the two gating strands at their periplasmic end in NgLptDE by 3 Å compared with SfLptDE and KpLtDE, with at least six and five hydrogen bonds between those strands, respectively (Fig. 4a–c). Luminal turn 1 preceding β1 adopts a conformation in NgLptDE that does not obstruct the luminal gate. Those residues of luminal turn 2 that are resolved in the cryo-EM map indicate an open conformation of this turn. This results in a direct connection between the hydrophobic groove of the N-terminal domain and the lumen of the β-barrel, which can be described as an opened luminal gate (Fig. 3a, b). The luminal gate has a diameter of approximately 10 Å as determined by the smallest distance between Leu250–Asp251 and Asp768–Leu769, which, combined with an opened lateral gate, could allow passage of the LPS core and O-antigen from the periplasmic space to the lumen of the barrel. Overall, the NgLptDE structure shows a wider diameter of the barrel lumen, reflecting a more open conformation of the luminal gate. With the distance between β1 and β26 being increased by 3 Å at the periplasmic side compare with SfLptD or KpLptD, this conformation represents a specific and until now unobserved stage of LPS transport.

### Disulfide bridges in NgLptDE and PMb-binding sites

The N-terminus in our LptD structure is not disulfide-bonded to the periplasmic turn between β24 and β25 as in SfLptDE and KpLptDE, albeit a corresponding cysteine is present. The N-terminal 63 residues (not including the signal sequence) are therefore likely flexible and not visible in the cryo-EM map. The N-terminal domain is fixed by a disulfide bridge to the barrel, but differently from known full-length structures of KpLptDE and SfLptDE (Supplementary Fig. 5c). The conserved two consecutive cysteines at the turn between β24 and β25 are shifted by one position and separated by a glycine in contrast to most LptD proteins (Supplementary Fig. 5c). Folding of LptD proteins involves several steps of disulfide bond reorganization of which ultimately one disulfide-bond is essential to connect the N-terminal jellyroll domain to the barrel^25^. Despite these differences, the orientation of the N-terminal domain was the same as in KpLptDE, whereas that of SfLptdE is rotated by approximately 20° (Fig. 3a).

PMb_21_ binds partly to the barrel rim and to extracellular loops 5, 11, 12, and 13 that all have been described as dispensable for LptD function^26^ (Supplementary Fig. 5a). PMb_51_ binds to the terminal β-strand of the jellyroll domain mainly via CDR3 that is forming a β-hairpin-like structure (β-strand augmentation) (Supplementary Fig. 5b). In the cell, this terminal strand of the jellyroll domain is deeply buried in the membrane, such that PMb_51_ or its parent Sb_51_ could not interfere with LPS transport by partial blockade of the exit path. Both Sb_21_ and Sb_51_ did not increase the susceptibility of *N. gonorrhoeae* toward vancomycin (Supplementary Fig. 7e). Further, neither of the Sbs bound to the surface of *E. coli* SF100 cells expressing NgLptDE (Supplementary Fig. 7f), which at least for Sb_51_ is not surprising due to the membrane localization of the epitope. Notably, paratopes like the CDR3 of PMb_51_ could also serve as template for the development of peptide antibiotics^11^. The PMbs did not lock LptDE in a specific conformation as we observed additional states in the sample (see below and Supplementary Fig. 8).

### Additional density observed in NgLptD

The 25 C-terminal residues of the LptD-barrel domain and luminal turn 2 were not visible in our initial cryo-EM map (“Overall” NgLptDE–PMb21–PMb51, Supplementary Table 1). In order to gain more insight into the function of the luminal gate in LPS transport, the cryo-EM data were reanalyzed focusing the refinement processing within a tight mask encompassing the N-terminal domain as well as the β1–β26 region of NgLptD only. The resulting cryo-EM map at 3.43 Å resolution allowed to trace the complete chain of the C-terminal region of LptD (Supplementary Fig. 6a–d). The C-terminal stretch following β26 extends deeply into the lumen of the β-barrel toward the restriction separating the two lobes of the barrel and in proximity to LptE. Interestingly, the C-terminal residues from Asn798 to Pro801 bind into the groove of the N-terminal jellyroll domain. Several salt bridges and hydrogen bonds stabilize the interaction (Supplementary Fig. 6d). Further work will be needed to determine if the observed position of the C-terminus could have a regulatory role similar to what has been suggested for the N-terminus^13^. In contrast to the crystal structures of SfLptD and KpLptDE, no helical region is observed in luminal turn 2.

### A fully opened LptD barrel from a subset of particles

**Fig. 5:**
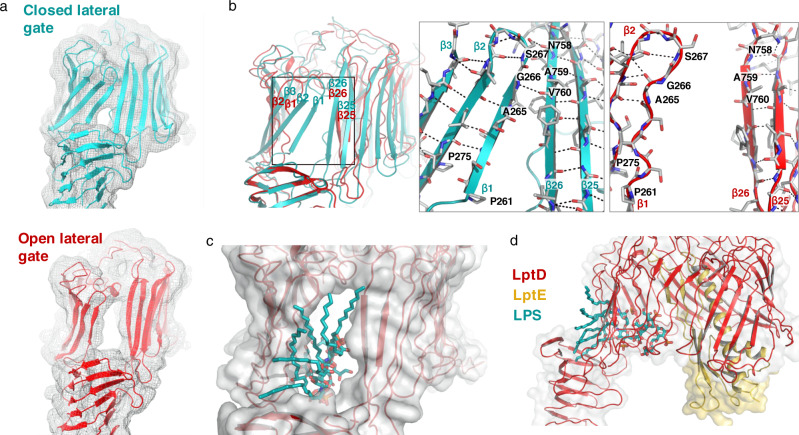
Comparison of the closed and open lateral gate of NgLptD. **a** Cryo-EM density of the NgLptD structures with closed (top) and open lateral gate (bottom) (maps displayed to the comparable contour level) with the respective model shown in cyan and red for the partly and fully open NgLptDE, respectively, (**b**) Superimposed models of NgLptD in partly (cyan) and fully open conformations (red). The boxes show the hydrogen-bond networks at the terminal strands of partly open (cyan, left) and fully open (red, right). **c** An LPS molecule (cyan) (extracted from 3FXI without modifications) fitted into the laterally open NgLptDE (red/yellow) viewed from extracellular or from the side (**d**).

Image processing assigned about one-fifth of the particles on the cryo-EM grid to a structure with an open barrel that did not fit the conformation of the main particle population. In order to exclude that this subpopulation could possibly result from the interaction with PMbs, we carefully analyzed two additional datasets obtained for NgLptDE and NgLptDE–PMb_21_. Interestingly, and even though high-resolution structures were not reachable, we observed in 2D classifications the same open and closed conformations (Supplementary Fig. 8). Both datasets for NgLptDE alone or in the presence of PMb_21_ (binding on the extracellular side of the barrel, away from the gate) present a mixture of open- and closed-conformation particles, thus ruling out the possibility of a structural artifact generated by the PMbs. Therefore, we used this subset of particles to compute a cryo-EM map of NgLptDE–PMb_21_–PMb_51_ with an open lateral gate at 4.72 Å resolution using a very tight mask (Figs. 4d, 5a–c). This map shows a full opening of the β-barrel devoid of H-bonds between β1 and β26, and a separation of ~10 Å between the extracellular ends and ~15 Å between the periplasmic ends of these strands. The diameter of the open barrel was not significantly different to the one in the partly open conformation. Only the six N-terminal and four C-terminal strands were shifted by more than 1.5 Å (Fig. 5b). This separation leads to a very large continuous solvent-accessible channel from the extracellular space through the barrel to the periplasm that could easily accommodate transiting LPS molecules (Fig. 5a). Docking of LPS into this experimental structure indicated that the saccharide portion of LPS likely enters the barrel and passes through it to the extracellular face, while lipid A would be inserted into the outer membrane (Fig. 5c, d). These data provide structural evidence for previously suggested events that lead to strand separation and are compatible with the idea of hydrogen-bond weakening between strands β1 and β26 through conserved prolines in β1 and β2^14^. Further, the delineated path of the lipid from LPS-cross-linking experiments is in accordance with the structure^27^. Consequently, we propose that the structure of a laterally fully opened LptD barrel could serve as a geometrical guideline for the design of macrocyclic antibiotics.

### A NgLptDE dimer mimics the principle of the Lpt trans-envelope complex

The analysis of the cryo-EM data revealed that a fraction of molecules is in a dimeric state with its interface at the periplasmic end of the N-terminal LptD jelly-roll domain (Supplementary Fig. 9a–d). The dimerization was already observed in SEC analysis and turned out to be rather stable (Supplementary Fig. 3a, b). In the crystal lattices of KpLptDE and SfLptDE, molecular contacts via the N-terminal domain of LptD were observed as well. However, in our cryo-EM structure, the dimer was displaying a pseudo-2-fold symmetry and generated a continuous groove between the two beta-jelly-roll domains. This further corroborates the oligomerization of jelly-roll domains of the Lpt-pathway members LptD, LptA, and LptC as a construction principle of the continuous hydrophobic slide across the periplasm^14^. Presumably due to mobility in the N-terminal domain, applying a C2 symmetry for dimeric particles did not result in higher resolution (Supplementary Table 1).

## Discussion

LPS transport requires a transient lateral opening of the LptD β-barrel to allow the synchronized translocation of the hydrophobic and hydrophilic portions of the large LPS molecules through the outer membrane^12–14^. For instance, cross-linking β-strands 1 and 26 through disulfide bridges abolished the function of LptD^12,16^, providing clear indications that these strands separate at least transiently for LPS release. The strands 1 and 26 do furthermore show less hydrogen bonds compared with other barrel structures, which support that these two strands are destined to temporarily separate. From mutagenesis studies and MD simulations, it was proposed that the two conserved prolines Pro230 and Pro245 in *Yersinia pestis* LptD (corresponding to Pro261 and Pro275 in NgLptD, respectively) (Fig. 4) play a crucial role in opening^14^ by attenuating the H-bonding between β1 and β26 to enable separation. Despite these clear indications, a lateral opening of the barrel was not fully evident from MD simulations^14^, or if so, only in extreme regimes^12^. Direct structural evidence for such a lateral opening was thus so far lacking. Using cryo-EM, we could find such a laterally opened structure that contributes to the completion of a full picture of LPS translocation. However, membrane proteins are susceptible to detergents used for solubilization to varying extent and this could alter their conformation. Thus, we can not rule out that barrel opening is potentiated or triggered by our selected detergent (see “Materials and methods”), e.g., by a weakening of the β1–β26 interaction. In this study, the choice of the detergent was guided by stability and purification-yield purposes to enable structural work on *N. gonorrhoeae* LptDE. Nevertheless, the fact that we observed this in only a subpopulation, and that even the closed conformation shows only few hydrogen bonds in comparison with earlier structures from other LptD transporters that stand in contrast to this idea. Due to the low resolution, we could achieve that for the open conformation, binding of detergent molecules cannot be confirmed or refuted either. Alternatively, NgLptDE might feature intrinsically less strong hydrogen-bonding between β1 and β26 (i.e., a higher open probability) in comparison with LptDE transporters from other species, leading to the subpopulation of fully opened barrels in our samples. Our comparison with PMb-complexed and uncomplexed NgLptDE rules out that the binders PMb21 and PMb51 caused artifactual opening as we observe open species also in uncomplexed NgLptDE particles (Supplementary Fig. 8). Altogether, our data suggest that the opening is a spontaneous event that does not strictly require a trigger; however, simulations by computational methods suggest that the presence of LPS in the N-terminal domain promotes strand separation (i.e., increases the open probability)^17^. The precise regulation of barrel-opening thus requires further investigation, also with respect to the role of LptE^28^ and the N-terminal domain of LptD^29^. In addition, future cryo-EM studies on LptDE transporters from other species and in different detergent-solubilized environment will help to resolve if barrel-opening is of different likelihood, depending on the species, generally a spontaneous event, or if it is promoted by LPS molecules or other factors.

Closed β-strand architectures such as barrels offer naturally rather shallow binding sites, to modulate their function. The most promising antibiotics to target these proteins are currently β-hairpin mimetics (constrained macrocyclic peptides such as Pol 7080/Murepavadin) that would bind to terminal beta-strands in open-barrel intermediates or to components of the Lpt trans-envelope complex^10,11,18,30^. Interestingly, thanatin, a naturally occurring antibiotically active peptide, targets to such β-strands of LptA, C, and D^29,31^. Non-hydrogen-bonded terminal β-strands, which eventually appear temporarily at the seam of laterally open barrels or at the termini of LptA/C in the Lpt pathway, can thus be understood as an Achilles’ heel of these architectures due to their propensity to bind peptides or mimetics thereof. On LptD, known β-hairpin peptides target the N-terminus of the jelly-roll domain as shown recently^29^. Taken together, the LptD structures of open-barrel conformations could be particularly well-suited to serve as template for the design of new antibiotics. The cleft between β1 and β26 leaves sufficient space to accommodate a peptidomimetic that could bind through hydrogen bonds to the LptD strands β1 and β26 (Supplementary Fig. 10). The cleft between β1 and β26 is an attractive ligand-binding location since a peptide could bind on two strands, thereby increasing binding strength and specificity. The cleft’s localization at the extracellular space allows to expand the possibilities for peptide design to include also hydrophilic, charged, and rather large residues, as it does not require the passage through the lipidic portion of the OM.

A backfolding of the N-terminal loop to the N-terminal domain of LptD has been described in a previous study on SfLptDE^13^. We observe for NgLptDE the C-terminus of LptD to be folded back to a very similar area on the N-terminal domain. It could thus be that LptD-protein termini are involved in the regulation of LPS transport. Such regulatory areas could represent another potentially interesting site for a drug interaction inhibiting the LPS transport^13,14^.

Small membrane proteins still impose a significant challenge for structural analysis by cryo-EM and even more so β-barrel proteins because of the lower contrast provided in comparison with α-helical architectures. In these cases, binders that serve as fiducial markers can greatly improve resolution, especially for structure-based drug design to further advance. To date, these chaperones are largely Fab fragments, but PMbs described here, represent an attractive alternative for cryo-EM.

Pro-Macrobodies (PMbs) were an essential tool to elucidate the structure of the drug target NgLptDE by cryo-EM to high resolution. The complexation with the PMbs had positive impact on several parameters of the structural analysis by cryo-EM. A better randomization of particle orientations contributes to the higher resolution of complexed compared with uncomplexed NgLptDE (Supplementary Fig. 1b,f) as was described for the GABA_A_ receptor in complex with megabodies^32,33^. For the EM analysis reported here, the major effect of PMbs is attributed to the increased size (230 kDa with PMbs vs. 110 kDa without) and the addition of distinct additional density to the particles for improved classification. Due to this enhanced particle selection and alignment, it was possible to reliably classify a subpopulation of particles and find the laterally open conformation of NgLptD. PMbs are different from the recently described megabodies^33^, as they feature only a single rigid linker. Our work provides evidence for a stable scaffold that significantly increased resolution of our target protein. Since any Nb or VHH can be converted to a PMb by fusing it in an identical rigid manner to the MBP moiety, they could become a widely applicable tool to improve the results in cryo-EM, especially for small and challenging targets. This development encourages the application of cryo-EM to complement X-ray crystallography for structure-based drug discovery of novel medicines.

## Methods

### Expression and purification of the *Ng*LptDE complex

*N. gonorrhoeae* wild-type full-length LptD (UniProt: Q5F651) and LptE (UniProt: Q5F9V6), with a hex-histidine tag on the C-terminus, were cloned into the expression vector pBAD22A (graciously provided by Professor Yihua Huang). NgLptDE complex was cotransformed in SF100 *E. coli* cells (graciously provided by Professor Yihua Huang). After transformation, preculture was started, and cells were grown overnight at 37 °C with 100 µg/ml ampicillin and 50 µg/ml kanamycin. Large LB-broth culture was then inoculated at a starting OD_600nm_ = 0.05 and grown at 37 °C with 100 µg/ml ampicillin. When OD_600nm_ = 0.8, temperature was switched to 20 °C and induction was performed at OD_600nm_ = 1 with 0.4% arabinose. After overnight induction at 20 °C, cells were harvested and resuspended in lysis buffer consisting of 200 mM NaCl, 50 mM Tris-HCl, pH 7.5, 10 mM MgCl_2_, 10 µg/ml DNAse I, 100 µg/ml AEBSF [4-(2-aminoethyl)benzenesulfonyl fluoride hydrochloride], and protease-inhibitor cocktail (Complete, Roche). Cells were cracked by multiple passages through a microfluidizer system using a pressure of 18,000 psi, and the lysate was centrifuged at 7500 × *g* for 10 min to remove the cell debris. The supernatant was collected, and inner membranes were solubilized by incubation with 2% Triton X-100 for 30 min at 4 °C under gentle agitation. The outer-membrane fraction was collected by centrifugation at 100,000 × *g* for 30 min at 4 °C. The pellet containing the outer-membrane fraction was resuspended in 200 mM NaCl, 50 mM Tris-HCl, pH 7.5, 20 mM imidazole, and 1% lauryl-dimethylamine oxide (LDAO), supplemented with protease-inhibitor cocktail (Complete, Roche), and incubated under gentle agitation for 12 h at 4 °C. Insoluble material was removed by centrifugation at 100,000 × *g* for 1 h at 4 °C. The supernatant was incubated in batches with ~5 ml NiNTA resin for 2 h at 4 °C under gentle agitation. The resin was subsequently washed by gravity flow with 10 column volumes (CV) of Wash buffer A (200 mM NaCl, 20 mM Tris-HCl pH 8.0, 20 mM imidazole, 1% LDAO), 10 CV of Wash buffer B (150 mM NaCl, 20 mM Tris-HCl, pH 8.0, 40 mM imidazole, and 0.5% LDAO), 10 CV of Wash buffer C (150 mM NaCl, 20 mM Tris-HCl, pH 8.0, 40 mM imidazole, and 0.2% LDAO), and 10 CV of Wash buffer D (150 mM NaCl, 20 mM Tris-HCl, pH 8.0, 40 mM imidazole, and 0.1% lauryl maltose neopentyl glycol (LMNG)). Elution was performed with 5 CV of Elution buffer (150 mM NaCl, 20 mM Tris-HCl pH 8.0, 300 mM imidazole, 0.01% LMNG). Eluted material was desalted against 150 mM NaCl, 20 mM Tris-HCl, pH 8.0, and 0.005% LMNG using disposable PD-10 desalting columns (GE Healthcare) and was concentrated in 100 kDa centrifugal concentrators (Millipore). Concentrated sample was further purified by SEC on a Superdex 200 Increase column, equilibrated to 150 mM NaCl, 20 mM Tris-HCl, pH 8.0, and 0.005% LMNG. The peak fractions corresponding to the monomeric or dimeric LptDE complex were kept separately, concentrated to ~1 mg/ml, and used for sybody generation, as well as biophysical and structural studies.

### Protein used for sybody generation and GCI characterization

*N. gonorrhoeae* (Zopf) Trevisan (ATCC 700825) LptD (1–801)-3C-His10 and LptE (1–159)–AVI complex was produced using *E. coli* SF100 cells and purified as described above, and an in vitro biotinylation step was added after the SEC step^34^. Briefly, LptDE protein was concentrated to 10 µM concentration and mixed with 40 µg of BirA *E. coli* enzyme, 5 mM ATP, 10 mM MgAc, and 15 µM biotin. The mixture was incubated for 16 h at 4 °C and a second SEC step was performed to desalt the sample and remove the BirA enzyme and free biotin in the following buffer: 20 mM Tris-HCl, pH 8.0, 150 mM NaCl, and 0.005% LMNG. Fractions corresponding to the monomeric complex peak were pooled, concentrated, and flash-frozen in liquid nitrogen for subsequent use.

### Sybody generation

Sybodies were generated as described previously^24,35^ with one notable difference. NgLptDE could not be produced in large amounts, which did not allow to use a large excess of nonbiotinylated NgLptDE for off-rate selection. Therefore, we pooled all the sybodies present after the first round of phage display and used them as competitors during the second round of phage display to perform an off-rate selection. To this end, the pDX_init plasmid outputs (of the concave, loop, and convex library) of the first round of phage display were purified by miniprep (Qiagen). FX cloning was performed to transfer the sybody pool from the pDX_init to the pSb_init expression plasmid using 2 µg of pDX_init pool and 1 µg of pSb_init. The cloning reaction was subsequently transformed into electrocompetent *E. coli* MC1061 cells (>10 mio cfu). The sybody pools were expressed as described for single sybodies using the pSb_init construct^35^. After expression of the pools in 600 ml cultures of TB medium, the sybodies were extracted from the cells by periplasmic extraction, purified by IMAC, and dialyzed overnight against Tris Buffered Saline (TBS). Precipitation was removed by centrifugation at 20,000 × *g* for 15 min. The pools were used at a concentration of approximately 100 µM to perform an off-rate selection for 2 min in the second round of phage display.

### Fluorescent labeling of sybodies

To perform site-specific labeling of the sybodies, a glycine located between SapI-restriction site and myc tag on the pSb_init backbone was mutated to cysteine via Quick change mutagenesis, thereby adding the following amino acids to the C-terminus of the sybody: GRACEQKLISEEDLNSAVDHHHHHH. The sybodies were expressed and purified as previously described^35^, except that 1 mM DTT was added to all buffers used for purification. Subsequently, DTT was removed and the sybody was rebuffered to degassed PBS using a PD10 desalting column and immediately mixing the sybody with Alexa Fluor 647 C_2_ maleimide (ThermoFisher Scientific) at a molar ratio of 1:3.6. The labeling reaction was carried out for 1 h at 4 °C. Excess label was removed by desalting the labeled sybody with a PD10 column.

### Cellular-binding assay

For cellular-binding assays, overnight cultures of *E. coli* SF100 cells with and without overexpression of NgLptDE were used. The number of cells was normalized by adjusting 1 ml of culture to an OD_600_ of 3. The cells were harvested by centrifugation washed three times with 500 µl of PBS containing 0.5% BSA (PBS–BSA), and subsequently blocked for 20 min in the same buffer. After an additional wash with 500 µl of PBS–BSA, the cells were incubated for 20 min in 100 µl of PBS–BSA containing 1 µM of the Alexa Fluor 647-labeled sybodies. After three washes with 500 µl of PBS, cells were resuspended in 100 µl of PBS and transferred to a microtiter plate with nontransparent walls. Fluorescence was measured in a plate reader with excitation of 651 nm and emission of 671 nm.

### Antibiotic-susceptibility assay

*N. gonorrhoeae* (Zopf) Trevisan (ATCC 700825) was streaked from a glycerol stock on blood agar and incubated for 24 h at 37 °C with 5% CO_2_ atmosphere. Colonies were scraped off the agar and resuspended in Fastidious broth at a density of McFarland 0.5^36^. The cells were further diluted 1:100 in Fastidious broth. In 96-well plates, dilution series of vancomycin with and without sybodies in Fastidious broth were prepared and mixed with the diluted culture. The plates were incubated without shaking at 37 °C with 5% CO_2_ atmosphere for 24 h. About 100 µl of a 0.04 mg/ml resazurin stock solution in PBS was added to the cells and incubated for one hour at 37 °C with 5% CO_2_ atmosphere. Fluorescence was measured at 571 nm excitation and 585 nm emission.

### Pro-Macrobody generation

Pro-Macrobodies (PMbs) were produced in *E. coli* as described earlier for the original macrobodies^20^. Briefly, two PCR-amplified fragments, the specific sybody and the C-terminal MBP, were cloned simultaneously into the expression vector pBXNPH3M (Addgene #110099)^37–39^ using FX cloning^40^. N-terminally of the resulting PMb insert, the plasmid expresses a pelB leader sequence followed by a deca-His tag, an MBP, and a 3C-protease site. The insert is fused during cloning through an overlapping proline-encoding CCG codon introduced by reverse and forward primers at the 3′- and 5′-end of the sybodies and MBP, respectively, and released by digestion with the type IIS restriction enzyme SapI (NEB). The second proline of the linker is encoded in the forward primer of the MBP (3′ of the overlapping CCG codon) and replaces the natural lysine. The resulting amino acid sequence of the linker is VTV***PP***LVI (VTV is the conserved C-terminus of sybodies, PP in italics/bold denotes the linker and underlined the truncated N-terminus of processed *E. coli* malE starting at Leu7). For the sybodies 21 and 51, to convert them into Pro-Macrobodies, forward primers 5′-TA TATAGCTCTTCTAGTCAGGTTCAGCTGGTTGAGAGCGGTGGTGGCC-3′ (Sb21), 5′-TATATAGCTCTTCT AGTCAAGTCCAGCTGGTGGAATCGGGTGGTGGTAG-3′ (Sb51), and reverse primers 5′-TATATA GCTCTTCTCGGCACAGTCACTTGGGTACCTTGG-3′ (Sb21) and 5′-TATATAGCTCTTCTCGGAACGGTAACTT GGGTGCCCTG-3′ (Sb51) were used. MBP for PMbs was amplified from pBXNPHM3 with forward primer 5′-TATATAGCTCTTCTCCGCCTCTGGTAATCTGGATTAACGG-3′ and reverse primer 5′-TATATAGCTCTTCTTGCA CCCGGAGTCTGCGCGTCTTTC-3′. The resulting amplicons for the sybodies and MBP, respectively, were mixed in a 1:1 molar ratio and cloned into pBXNPHM3. PMbs were expressed in terrific broth in MC1061 *E. coli* cells at 37 °C by induction with 0.02% arabinose at an OD_600_ = 0.7. After 3.5 h, cells were harvested and resuspended in lysis buffer consisting of 150 mM NaCl, 50 mM Tris-HCl pH 8, 20 mM imidazole, 5 mM MgCl_2_, 10% glycerol, 10 µg/ml DNAse I, and protease inhibitors (Complete, Roche). Cells were cracked and the lysate was centrifuged for 30 min at 147,000 × *g* in a Beckman 45Ti rotor. Subsequently, the supernatant was incubated in batch with ~3 ml NiNTA resin/6 L of culture. The resin was washed with 150 mM KCl, 40 mM imidazole, pH 7.6, and 10% glycerol and eluted with 150 mM KCl, 300 mM imidazole, pH 7.6, and 10% glycerol. The N-terminal MBP with the deca-His tag was removed by cleavage with 3 C protease overnight during dialysis against 150 mM KCl, 10 mM Hepes–NaOH, 20 mM imidazole, pH 7.6, and 10% glycerol. After removal of the His-tagged MBP by Re-IMAC, the unbound material was concentrated in 50 kDa centrifugal concentrators (Millipore) and subjected to SEC on a Superdex 200 Increase column (equilibrated to 150 mM NaCl, 10 mM Hepes–Na, pH 7.6) connected to an AKTA system (GE) operated with Unicorn software v7.1. The eluted fractions with the PMbs were supplemented with 20% glycerol, concentrated to 3–8 mg/ml, and aliquots were flash-frozen in liquid nitrogen for subsequent use. Macrobody versions of sybodies 21 and 51 with the original VK-linker were expressed and purified in the same way.

### SEC analysis/purification of LptDE–PMb complexes

To identify ternary and quaternary complexes of NgLptDE with various PMbs against NgLptDE, monomeric NgLptDE was first separated from dimers by SEC on a Superdex 200 Increase 10/300 column in 150 mM NaCl, 20 mM Tris-Cl, pH 8, and 0.005% LMNG. A complex was formed by addition of 3- to 4-fold molar excess of PMb51 to NgLptDE sample. The uncomplexed and PMb-bound samples were analyzed on an Agilent 1260 Infinity II HPLC operated with Chemstation 2.12.26 using a Superdex 200 Increase 5/150 column and Trp-fluorescence detection after 10–15 min incubation time on ice. Binding was indicated by a shift to earlier elution volumes and increase in the UV absorption and Trp fluorescence. The quaternary complex was formed by adding PMb21 in 3- to 4-fold molar excess to the preformed NgLptDE–PMb51 complex. The quaternary NgLptDE–PMb51–PMb21 complex showed the most distinct shift and fluorescence increase and was therefore chosen for scale-up and cryo-EM analysis.

### Binding analytics by grating coupled interferometry

Initial screen with ELISA-positive sybodies was performed at 20 °C on the Wave delta instrument from Creoptix in Tris 25 mM pH 7.5, NaCl 300 mM, and DDM 0.1% as running buffer. Biotinylated protein was immobilized on a 4PCP-S (streptavidin) chip, conditioned with 1 M NaCl, 0.1 M sodium borate, at levels between 600 and 700 pg/mm2 to avoid any mass-transport limitation. One injection of each sybody (200 nM) was performed and binding responses were evaluated with the Wave control software. The ten best sybodies in terms of slower K_off_ as well as quality of binding signals obtained were characterized further with dose–response analysis in order to determine accurate kinetic parameters. For each ten-sybody, 8 concentrations were recorded (serial 2-fold dilution) in duplicate injections, and equilibrium as well as kinetic data were analyzed and fitted with 1:1 model. Fits were of high quality (black curves) and recapitulate the experimental data (red curves). At higher concentration of sybodies, bulk (RI) effects could be observed, but this effect did not influence data analysis. PMb21 showed a slightly decreased affinity (3 to 4-fold) compared with the respective sybody 21. This loss can be explained by a faster K_off_ of the PMb21.

### Sample preparation and cryo-EM data acquisition

Quantifoil (1/2) 200-mesh copper grids were glow-discharged for 20 sec prior to sample freezing. About 3 µl of NgLptDE–PMb51–PMb21 complex at a concentration of 1 mg/ml were placed on the grid, blotted for 3.0 s, and flash-frozen in a mixture of liquid propane and liquid ethane cooled with liquid nitrogen using a Vitrobot Mark IV (FEI) operated at 4 °C and under 100% humidity.

The EM data-collection statistics in this study are reported in Supplementary Table 1. Data were recorded on a FEI Titan Krios transmission-electron microscope, operated at 300 kV and equipped with a Quantum-LS energy filter (slit width 20 eV, Gatan Inc.) containing a K2 Summit direct electron detector. Data were automatically collected using the software SerialEM^41^. Dose-fractionated exposures (movies) were recorded in electron-counting mode, applying 60 electrons per square angstrom (e^−^/Å^2^) over 45 frames, or 50 e^−^/Å^2^ over 35 frames for, respectively, the NgLptDE (apo) or the NgLptDE–PMb51–PMb21 samples. A defocus range of −0.8 to −2.8 µm was used and the physical pixel size was 0.64 Å/pixel for the NgLptDE and 0.82 Å/pixel for the NgLptDE–PMb51–PMb21 datasets. Recorded data were online-analyzed and preprocessed using FOCUS^42^, which included gain normalization, motion correction, and calculation of dose-weighted averages with MotionCor2^43^, as well as estimation of micrograph defocus with CTFFIND4^44^.

### Image processing

The following processing workflows were used for the samples in the study. The aligned movies were imported into CryoSPARC V2^45^. A set of aligned averages with a calculated defocus range of −0.6 to –3.0 μm was selected from which averages with poor CTF-estimation statistics were discarded. Automated particle picking in CryoSPARC V2 resulted in 815,057 particle locations for the NgLptDE–PMb51–PMb21 sample. After several rounds of 2D classification, 490,743 particles were selected and subjected to 3D classification using the multi class ab initio refinement process (5 classes, 0.4 similarity) and heterogeneous refinement. The best-resolved class consisting of 184,206 particles was finally subjected to 3D nonuniform refinement. The overall resolution of the resulted map was estimated at 3.40 Å based on the Fourier shell correlation (FSC) at 0.143 cutoff^46^. To visualize the LptD–NTD-dimerization interface, another round of 3D heterogeneous refinement was performed and a subset consisting of 80,140 particles was selected. Those particle coordinates used re-extraction of particle images with an increased box size. Particles were recentered by 2D classification in order to process the dimeric LptDE complex. Ab initio reconstruction and nonuniform refinement on this set of particles resulted in a _DIMER_NgLptDE–PMb51–PMb21 map with an overall resolution of 5.27 Å. As for the opened state, a multiclass ab initio refinement process and heterogeneous refinement was performed for the NgLptDE–PMb51–PMb21 sample. Of the five 3D classes, one class consisting of 93,151 particles was further refined by computationally removing the density corresponding to the detergent micelle with the particle-subtraction tool within CryoSPARC V2, followed by 3D local refinement. The resulting map had an estimated overall resolution of 4.72 Å as judged by FSC at 0.143 cutoff. Analysis of the apo NgLptDE was performed similarly to the NgLptDE-PMb21-PMb51. Briefly, a set of aligned averages with a calculated defocus range of −0.6 to –3.0 μm was selected, from which averages with poor CTF-estimation statistics were discarded. Automated particle picking in CryoSPARC V2 resulted in 1,395,392 particle locations for the NgLptDE. After several rounds of 2D classification, 196,182 particles were selected and subjected to 3D classification using the multiclass ab initio refinement process (3 classes, 0.1 similarity) and heterogeneous refinement for the 2 best classes. The best-resolved class consisting of 119,115 particles was finally subjected to 3D nonuniform refinement. The overall resolution of the resulted map was estimated at 4.6 Å based on the Fourier shell correlation (FSC) at 0.143 cutoff.

### Model building and refinement

An initial LptDE model was generated using SWISS-MODEL^47^, using as templates the KpLptD structure (PDB-ID 5IV9) and the EcLptE structure (PDB-ID 4RHB). The template for building the PMb_21_ coordinates into the EM map was based on the X-ray structure that was solved specifically for this study (PDB-ID 7OMT). The same structure was also used to model the maltose-binding protein region of PMb_51_.

Rigid-body fitting was initially done in Chimera^48^ followed by manually rebuilding of the model in Coot^49^. The remaining clashes between sidechains were detected using Schrodinger version 2019-4 (Maestro, Schrödinger, LLC, New York, NY, 2021), and remodeled using prime^50^. Manual inspection of missing H-bonds in the model was used to refine sidechain positions. Finally, real-space refinement was performed in Phenix version 1.17–3644, applying Ramachandran plot restraints^51^.

### Molecular-dynamics simulations

Possible linkers connecting the VHH to MBP were assessed by all-atom MD simulations. After 300 ns of equilibration, conformational space was explored by conducting 500 ns MD trajectories initiated from a structure solved with a (Val122 and Lys123) VL linker (PDB-ID 6HD8), as well as the same set of coordinates, but modeled with an engineered (Pro122–Pro123) PP linker. Both trajectories were later aligned on the VHH structure (residues 1–120) to show the larger conformational flexibility of the VK linker. The macrobody interdomain (VHH to MBP) angle was also monitored during MD trajectories. This angle was defined as the angle between the geometrical centers of residue 1–120 (Nb), residue 122–123 (linker), and residue 124–486 (MBP). All MD simulations were conducted in explicit water, at room temperature (300 K, or 26.85 °C), and with Desmond standard parameterization^52^ and the OPLS3e force field^53^.

### Crystallization and structure determination of PMb_21_

Purified PMb_21_ in 150 mM NaCl, 10 mM HEPES–Na, pH 7.5, concentrated to 10 mg/ ml and supplemented with 2.5 mM D-(+)-maltose, was subjected to crystallization screens. The protein crystallized in 0.2 M MgCl_2_, 0.1 M Hepes, pH 7.5, and 30% PEG400. For cryoprotection, crystals were soaked in 0.2 M MgCl_2_, 0.1 M Hepes, pH 7.5, 35% PEG400, and 2.5 mM D-(+)-maltose. Crystals were flash-frozen in liquid N_2_ and data were collected at the PXII (X10SA) beamline of the Swiss Light Source (SLS, Villigen).

The dataset was integrated and scaled with XDS (built=20190806). The structure was solved by PHASER version 2.8.3 (CCP4 7.0.077) using MBP (PDB 1N3W) and a nanobody (PDB 5FWO) as molecular replacement-search model. The structure was initially refined using REFMAC version 5.8.0257 (CCP4 7.0.077) and later with PHENIX version 1.16, together with iterative model building in COOT using 2FoFc and FoFc map.

### Figure preparation

Figures were prepared using the programs Chimera X (http://www.rbvi.ucsf.edu/chimerax/^54^), Chimera (http://www.cgl.ucsf.edu/chimera/^48^), PyMOL (http://www.pymol.org/) and Schrödinger (www.schrodinger.com).

### Reporting summary

Further information on research design is available in the Nature Research Reporting Summary linked to this article.

## Supplementary information

Supplementary Information

Reporting Summary

## Data Availability

The EM map for the high-resolution NgLptDE–PMb_21_/PMb_51_ complex was deposited in the Electron Microscopy Data Bank under accession code EMD-12990. Atomic coordinates for NgLptDE from the cryo-EM study were deposited in the Protein Data Bank under accession code PDB-7OMM. The X-ray structure of PMb_21_ was deposited with accession codes PDB-7OMT. All other data are available from the corresponding authors upon reasonable request. Source data are provided with this paper.

## Source data

Source Data

## References

[1] Centers for Disease Control and Prevention. *Antibiotic resistance threats in the United States* (2019).

[2] Bertani, B. & Ruiz, N. Function and Biogenesis of Lipopolysaccharides. *EcoSal Plus*, 10.1128/ecosalplus.ESP-0001-2018 (2018). 10.1128/ecosalplus.esp-0001-2018 PMC6091223 30066669

[3] Narita S, Tokuda H (2009). Biochemical characterization of an ABC transporter LptBFGC complex required for the outer membrane sorting of lipopolysaccharides. FEBS Lett..

[4] Freinkman E, Chng SS, Kahne D (2011). The complex that inserts lipopolysaccharide into the bacterial outer membrane forms a two-protein plug-and-barrel. Proc. Natl Acad. Sci. USA.

[5] Freinkman E, Okuda S, Ruiz N, Kahne D (2012). Regulated assembly of the transenvelope protein complex required for lipopolysaccharide export. Biochemistry.

[6] Whitfield C, Stephen Trent M (2014). Biosynthesis and export of bacterial lipopolysaccharides. Annu. Rev. Biochem..

[7] Ruiz N, Gronenberg LS, Kahne D, Silhavy TJ (2008). Identification of two inner-membrane proteins required for the transport of lipopolysaccharide to the outer membrane of Escherichia coli. Proc. Natl Acad. Sci. USA.

[8] Lehman, K. M. & Grabowicz, M. Countering gram-negative antibiotic resistance: recent progress in disrupting the outer membrane with novel therapeutics. *Antibiotics* **8**, 163 (2019). 10.3390/antibiotics8040163 PMC6963605 31554212

[9] Wu T (2006). Identification of a protein complex that assembles lipopolysaccharide in the outer membrane of Escherichia coli. Proc. Natl. Acad. Sci. USA.

[10] Srinivas N (2010). Peptidomimetic antibiotics target outer-membrane biogenesis in pseudomonas aeruginosa. Science.

[11] Zerbe K, Moehle K, Robinson JA (2017). Protein epitope mimetics: from new antibiotics to supramolecular synthetic vaccines. Acc. Chem. Res..

[12] Dong H (2014). Structural basis for outer membrane lipopolysaccharide insertion. Nature.

[13] Qiao S, Luo Q, Zhao Y, Zhang XC, Huang Y (2014). Structural basis for lipopolysaccharide insertion in the bacterial outer membrane. Nature.

[14] Botos I (2016). Structural and functional characterization of the LPS transporter LptDE from gram-negative pathogens. Structure.

[15] Botos, I., Noinaj, N. & Buchanan, S. K. Insertion of proteins and lipopolysaccharide into the bacterial outer membrane. *Philos. Trans. R. Soc. B Biol. Sci*. **372**, 20160224 (2017). 10.1098/rstb.2016.0224 PMC5483524 28630161

[16] Gu Y (2015). Lipopolysaccharide is inserted into the outer membrane through an intramembrane hole, a lumen gate, and the lateral opening of lptd. Structure.

[17] Lundquist KP, Gumbart JC (2020). Presence of substrate aids lateral gate separation in LptD. Biochim. Biophys. Acta Biomembr..

[18] Luther A (2019). Chimeric peptidomimetic antibiotics against Gram-negative bacteria. Nature.

[19] Remaut H, Waksman G (2006). Protein-protein interaction through β-strand addition. Trends Biochem. Sci..

[20] Brunner JD (2020). Structural basis for ion selectivity in TMEM175 K^+^ channels. Elife.

[21] Hite RK, MacKinnon R (2017). Structural Titration of Slo2.2, a Na^+^-Dependent K^+^ Channel. Cell.

[22] Frank J (2018). New opportunities created by single-particle Cryo-EM: the mapping of conformational space. Biochemistry.

[23] Hofmann S (2019). Conformation space of a heterodimeric ABC exporter under turnover conditions. Nature.

[24] Zimmermann I (2018). Synthetic single domain antibodies for the conformational trapping of membrane proteins. Elife.

[25] Ruiz N, Chng SS, Hinikera A, Kahne D, Silhavy TJ (2010). Nonconsecutive disulfide bond formation in an essential integral outer membrane protein. Proc. Natl Acad. Sci. USA.

[26] Storek KM (2019). Massive antibody discovery used to probe structure–function relationships of the essential outer membrane protein LptD. Elife.

[27] Li X, Gu Y, Dong H, Wang W, Dong C (2015). Trapped lipopolysaccharide and LptD intermediates reveal lipopolysaccharide translocation steps across the *Escherichia coli* outer membrane. Sci. Rep..

[28] Malojčić G (2014). LptE binds to and alters the physical state of LPS to catalyze its assembly at the cell surface. Proc. Natl Acad. Sci. USA.

[29] Fiorentino F (2021). Dynamics of an LPS translocon induced by substrate and an antimicrobial peptide. Nat. Chem. Biol..

[30] Robinson JA (2019). Folded synthetic peptides and other molecules targeting outer membrane protein complexes in Gram-negative bacteria. Front. Chem..

[31] Vetterli, S. U. et al. Thanatin targets the intermembrane protein complex required for lipopolysaccharide transport in *Escherichia coli*. *Sci. Adv*. **4**, (2018). 10.1126/sciadv.aau2634 PMC6235536 30443594

[32] Laverty D (2019). Cryo-EM structure of the human α1β3γ2 GABAA receptor in a lipid bilayer. Nature.

[33] Uchański T (2021). Megabodies expand the nanobody toolkit for protein structure determination by single-particle cryo-EM. Nat. Methods.

[34] Kuhn, B. T. et al. In: *Expression, purification, and structural biology of membrane proteins* Vol. 2127 (eds Perez, C., & Maier, T.) 151–165 (Humana, 2020).

[35] Zimmermann I (2020). Generation of synthetic nanobodies against delicate proteins. Nat. Protoc..

[36] Jacobson RK, Notaro MJ, Carr GJ (2019). Comparison of Neisseria gonorrhoeae minimum inhibitory concentrations obtained using agar dilution versus microbroth dilution methods. J. Microbiol. Methods.

[37] Ehrnstorfer IA, Geertsma ER, Pardon E, Steyaert J, Dutzler R (2014). Crystal structure of a SLC11 (NRAMP) transporter reveals the basis for transition-metal ion transport. Nat. Struct. Mol. Biol..

[38] Geertsma ER (2015). Structure of a prokaryotic fumarate transporter reveals the architecture of the SLC26 family. Nat. Struct. Mol. Biol..

[39] Schenck S (2017). Generation and characterization of anti-VGLUT nanobodies acting as inhibitors of transport. Biochemistry.

[40] Geertsma ER, Dutzler R (2011). A versatile and efficient high-throughput cloning tool for structural biology. Biochemistry.

[41] Mastronarde DN (2005). Automated electron microscope tomography using robust prediction of specimen movements. J. Struct. Biol..

[42] Biyani N (2017). Focus: the interface between data collection and data processing in cryo-EM. J. Struct. Biol..

[43] Zheng SQ (2017). MotionCor2: anisotropic correction of beam-induced motion for improved cryo-electron microscopy. Nat. Methods.

[44] Rohou A, Grigorieff N (2015). CTFFIND4: fast and accurate defocus estimation from electron micrographs. J. Struct. Biol..

[45] Punjani A, Rubinstein JL, Fleet DJ, Brubaker MA (2017). CryoSPARC: algorithms for rapid unsupervised cryo-EM structure determination. Nat. Methods.

[46] Scheres SHW, Chen S (2012). Prevention of overfitting in cryo-EM structure determination. Nat. Methods.

[47] Waterhouse A (2018). SWISS-MODEL: homology modelling of protein structures and complexes. Nucleic Acids Res..

[48] Pettersen EF (2004). UCSF Chimera - a visualization system for exploratory research and analysis. J. Comput. Chem..

[49] Emsley P, Lohkamp B, Scott WG, Cowtan K (2010). Features and development of Coot. Acta Crystallogr. Sect. D Biol. Crystallogr..

[50] Zhu K (2014). Docking covalent inhibitors: a parameter free approach to pose prediction and scoring. J. Chem. Inf. Model..

[51] Liebschner D (2019). Macromolecular structure determination using X-rays, neutrons and electrons: Recent developments in Phenix. Acta Crystallogr. Sect. D Struct. Biol..

[52] Bowers, K. J. et al. In *Proceedings of the 2006 ACM/IEEE**Conference on Supercomputing* (ACM/IEEE, 2006).

[53] Harder E (2016). OPLS3: a force field providing broad coverage of drug-like small molecules and proteins. J. Chem. Theory Comput..

[54] Pettersen EF (2021). UCSF ChimeraX: structure visualization for researchers, educators, and developers. Protein Sci..

