# Supplementary material for: Cryo-EM structures of a LptDE transporter in complex with Pro-macrobodies offer insight into lipopolysaccharide translocation
Source: Nat Commun. 2022 Apr 5;13:1826. doi: 10.1038/s41467-022-29459-2 (PMC8983717; doi:10.1038/s41467-022-29459-2)
Supplement: Supplementary file 1 — Supplementary Information [file 41467_2022_29459_MOESM1_ESM.pdf]

Supplementary Information

Cryo-EM structures of a LptDE transporter in complex with Pro-macrobodies offer insight into lipopolysaccharide translocation

Mathieu Botte[1†], Dongchun Ni[2†], Stephan Schenck[1‡†], Iwan Zimmermann[3§], Mohamed Chami[2], Nicolas Bocquet[1], Pascal Egloff[3§], Denis Bucher[1], Matilde Trabuco[1], Robert K.Y. Cheng[1], Janine D. Brunner[4,5], Markus A. Seeger[3], Henning Stahlberg[2] and Michael Hennig[1*]

1 leadXpro AG, Park Innovaare, 5234 Villigen, Switzerland

2 C-CINA, Biozentrum, University of Basel, Mattenstr. 24, 4058 Basel, Switzerland

3 Institute of Medical Microbiology, University of Zürich, Gloriastasse 28/30, 8006 Zürich

4 Laboratory of Biomolecular Research, Division of Biology and Chemistry, Paul Scherrer Institute (PSI), 5232 Villigen, Switzerland

5 VIB-VUB Center for Structural Biology, VIB, 1050 Brussels, Belgium

‡ Present address: VIB-VUB Center for Structural Biology, VIB, 1050 Brussels, Belgium

§ Present address: Linkster Therapeutics AG, 8006 Zürich

† Authors contributed equally

*Corresponding author

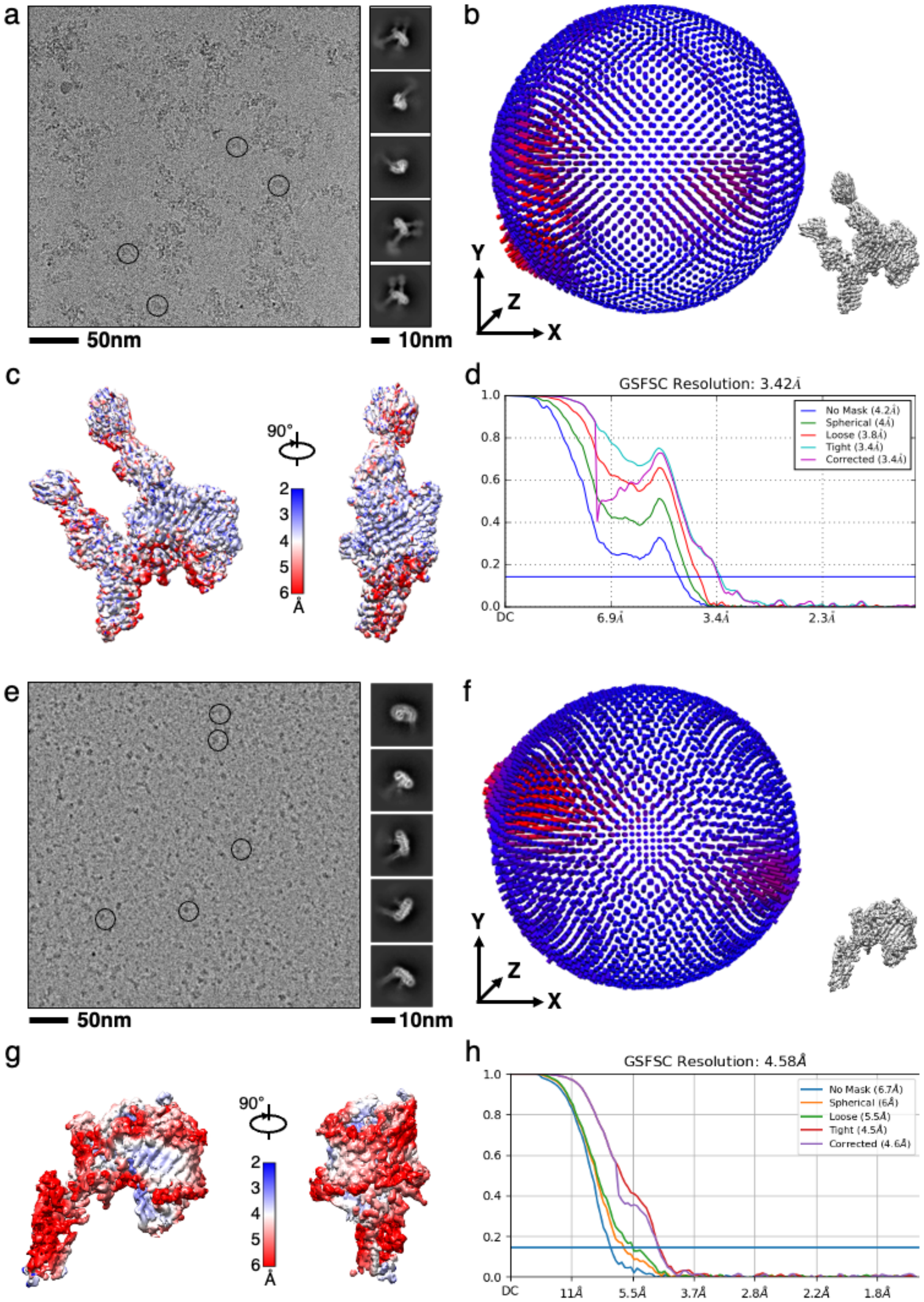

Supplementary Fig. 1: Cryo-EM acquisition and maps of complexed and uncomplexed NgLptDE. (a) Example micrograph of vitrified NgLptDE-PMb21/PMb51 quaternary complex. Representative particles are circled in black. Class averages are shown on the right. (b) Distribution of the particle views that were used to build the map projected on a sphere relative to the orientation shown on the lower right. (c) Map of the laterally closed NgLptDE-PMb21/PMb51 quaternary complex with color-coded local resolution (middle panel). (d) Gold standard Fourier-Shell correlation resolution plot with a cutoff at 0.143 indicated by a dashed line. (e-h) Respective data as in (a-d) for the uncomplexed NgLptDE dataset. Source data are provided as a Source Data file.

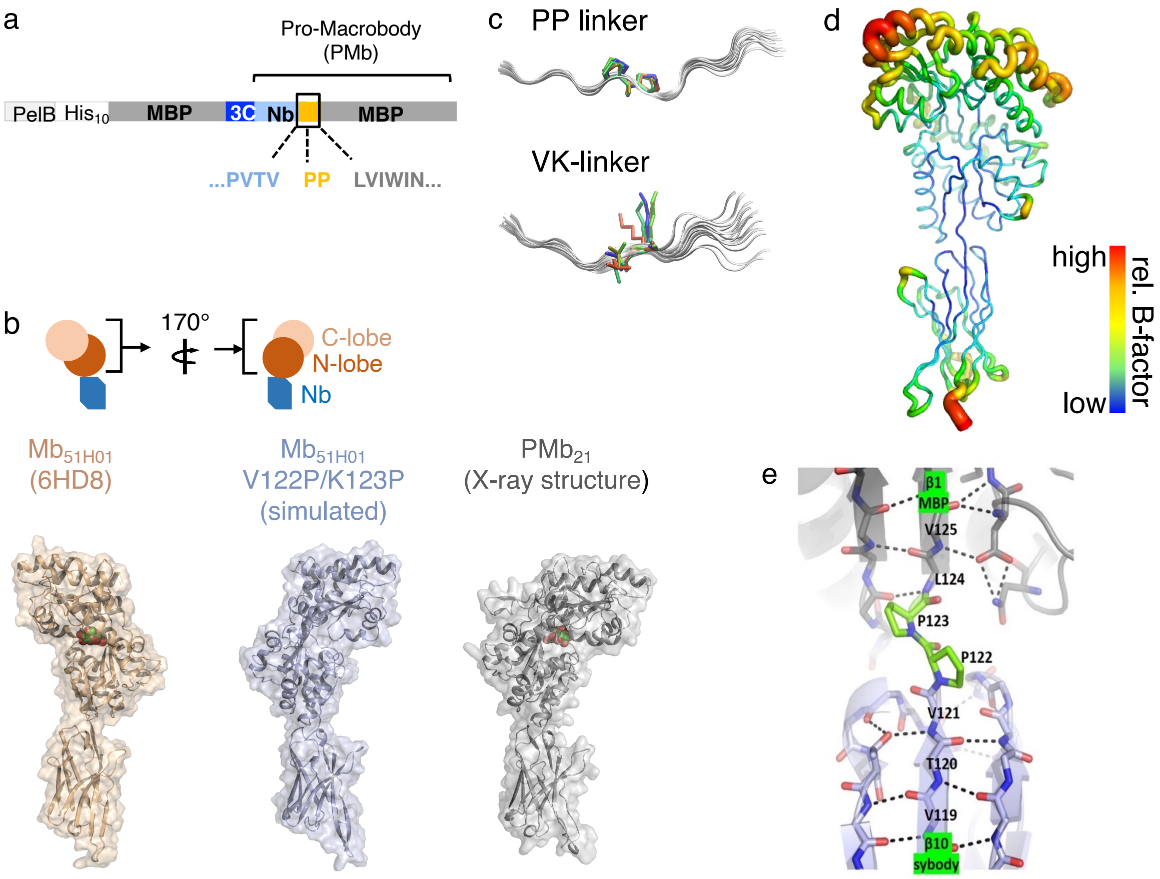

Supplementary Fig. 2: Design and properties of Pro-Macrobodies. (a) Construct layout for the expression of PMbs. (b) Comparison of the structures of the template Mb_{51H01} (left), a frame from the simulated (in-silico mutated) Mb_{51H01}(middle) and the PMb_{21} X-ray structure. The orientation of the MBP moiety in Mb_{51H01} and its mutated version relative to the Nb-moiety is shown on top. Maltose is shown as space-fill model in the left and right panels. (c) C-alpha traces of the linker region in the MD simulations for the two types of linker. Single frames of the MD simulations were stacked, and the linker residues are shown as sticks. (d) Relative B-factors of the PMb_{21} crystal structure shown as putty in rainbow colors. (e) Hydrogen bonds around the linker region of PMb_{21} indicated by black dashes. The connected β-strands of the sybody (β10) and MBP (β1) are indicated together with the linker residues. The newly introduced residues P122/123 are labelled green. The C-terminal end of the sybody and the N-terminal part of MBP are dominated by β-sheets that are interrupted by the intrinsically rigid di-proline motif.

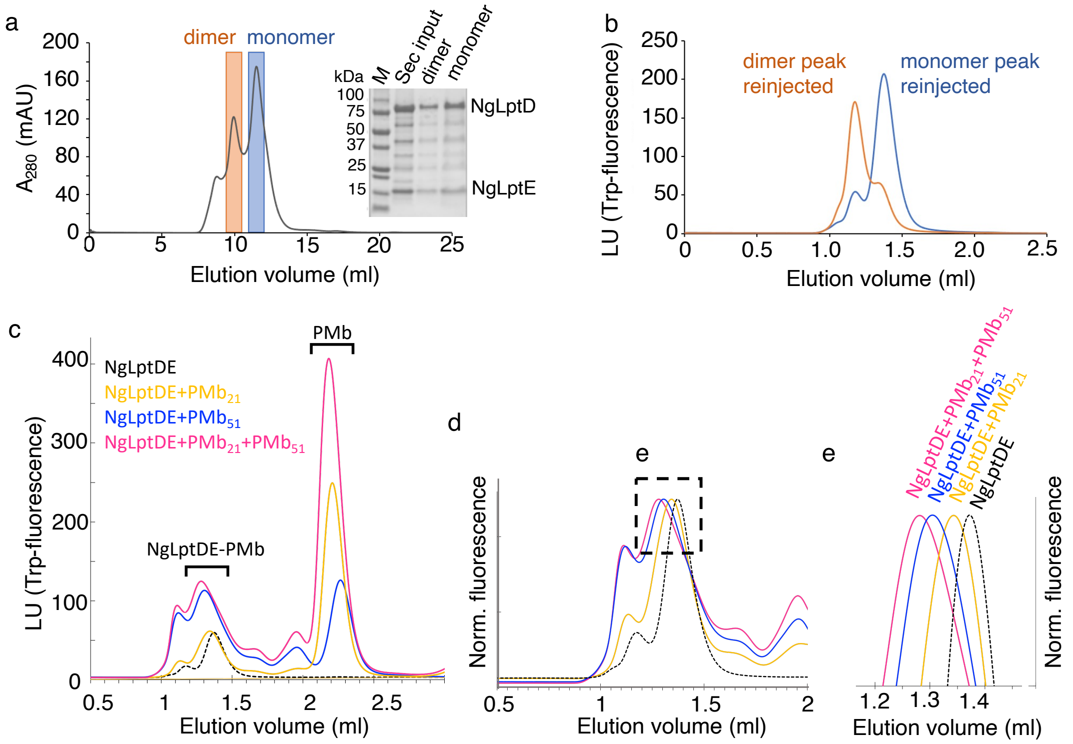

Supplementary Fig. 3: Size exclusion chromatography of NgLptDE and NgLptDE-PMb complexes. (a) Representative elution profile of dialysed and concentrated NgLptDE after IMAC subjected to a Superdex 200 10/300 Increase column. Dimer and Monomer peaks are indicated in orange and blue. A representative Coomassie stained SDS gel is shown as inset with LptD and LptE indicated. (b) Reinjection of the peak fractions with dimeric and monomeric NgLptDE to a Superdex 200 5/150 column. (c) NgLptDE mixed with excess of PMbs and subjected to a Superdex 200 5/150 column. Elution was monitored by Trp-fluorescence. (d,e) Enlarged and normalized peaks of NgLptDE and its complexes with PMbs in (c). Source data are provided as a Source Data file.

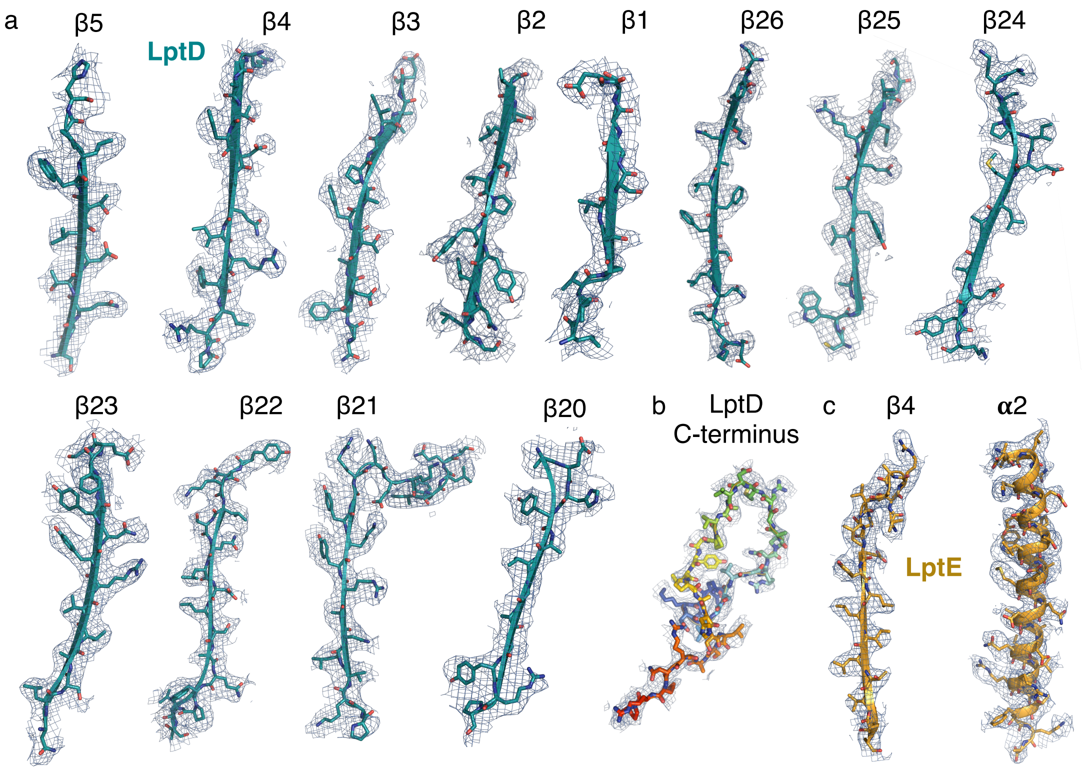

Supplementary Fig. 4: Agreement of the NgLptDE-PMb$_{21}$-PMb$_{51}$ Cryo-EM map and the corresponding model. (a) β-strands of the NgLptD barrel shown with the cryo-EM map contoured at 9σ with the model superimposed. (b) LptD C-terminus colored in rainbow from N-to C-terminal ends. (c) β-strand 4 and α-helix 2 of LptE.

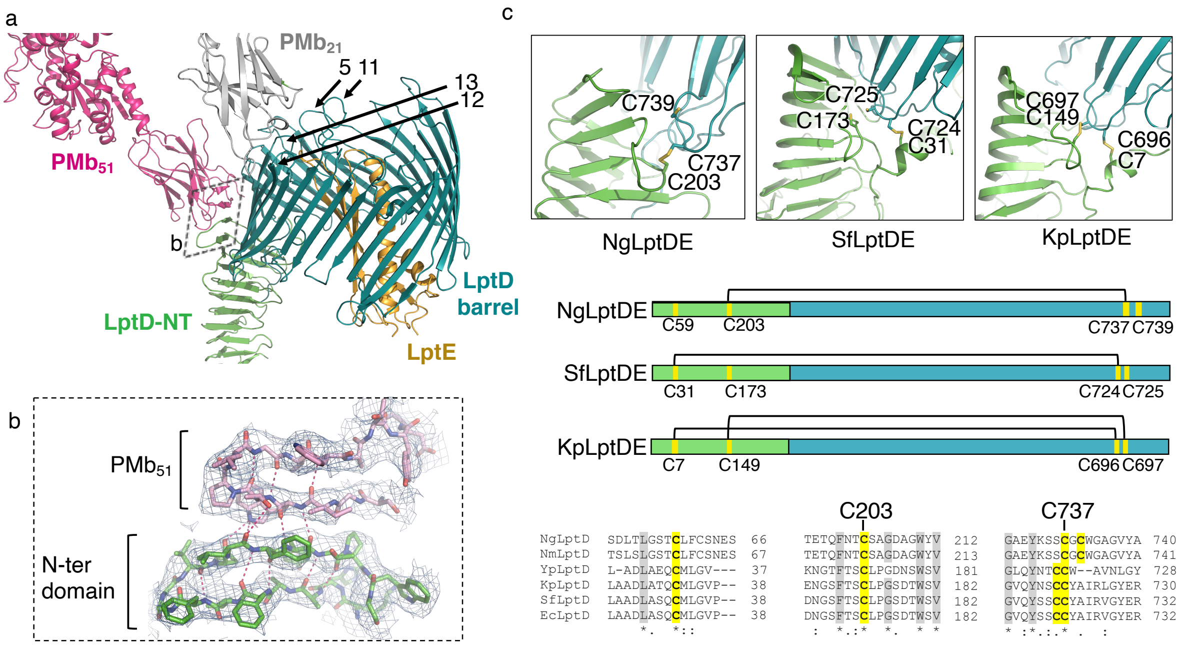

Supplementary Fig. 5: Details of the PMb51 CDR3 binding and disulfide bridges in LptD. (a) Overview of the binding regions of PMb_{21} and PMb_{51} at LptD barrel-loops and N-terminal domain. (b) View from the lateral seam in the LptD barrel (boxed in (a)) onto the edge of the N-terminal domain showing the hydrogen bonds between CDR3 and the terminal strand of the N-terminal domain and intramolecular bonds of CDR3. (c) Disulfide bridge patterns of conserved cysteines in LptD proteins from different bacterial species. The scheme in the middle shows the disulfide bridges schematically. An alignment (bottom) shows that the cysteines at the C-terminal end of *Neisseria* species are spaced by a glycine and thus different from other gram-negative bacteria.

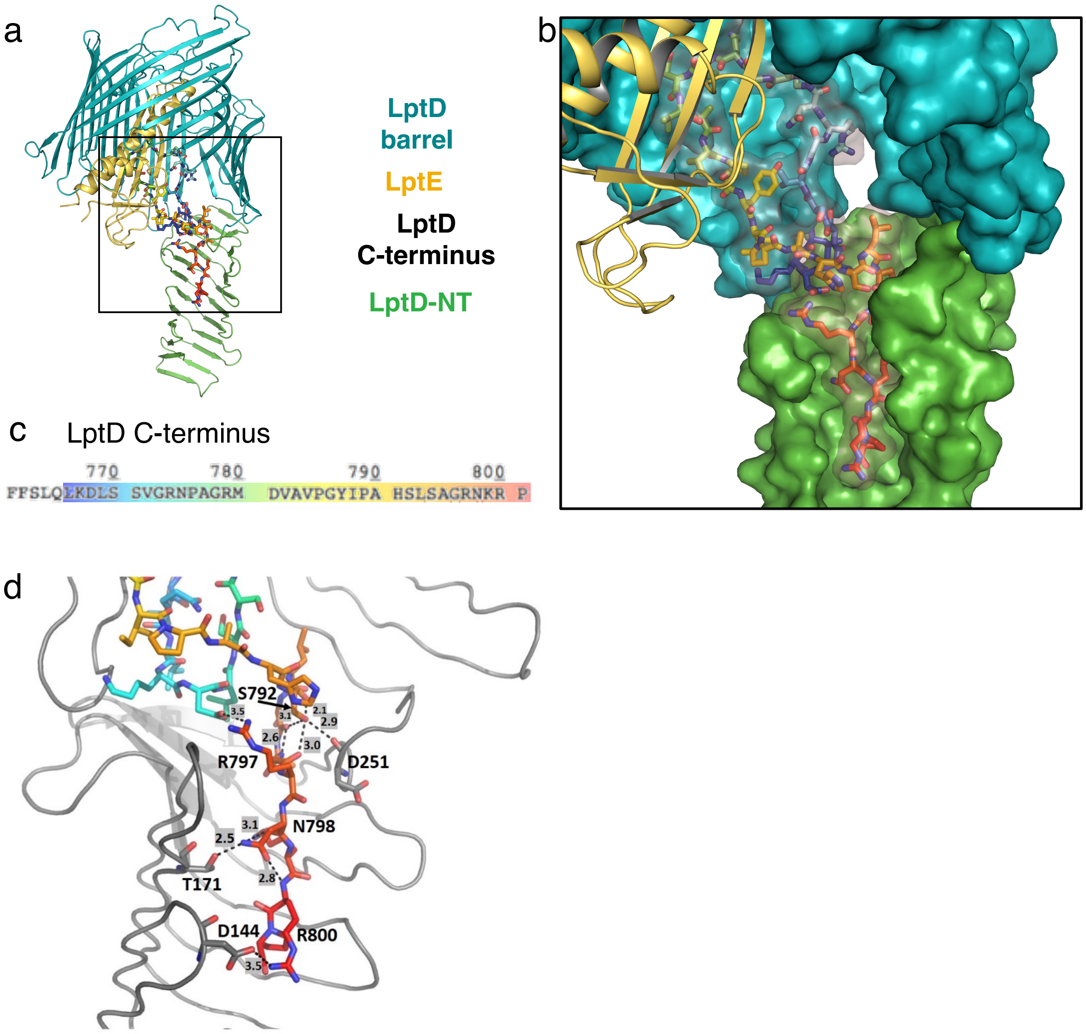

Supplementary Fig. 6: Binding of the C-terminus of NgLptD to the N-terminal jellyroll domain. (a) Overview of the location of the C-terminus in NgLptD in the complex. C-terminal residues are shown as sticks in rainbow colors as depicted in (c). (b) Close-up view of the C-terminus in the N-terminal domain in space fill model (transparent) with residues shown as sticks in rainbow colors. (c) Color code for the C-terminus used in (a,b,d) (d) Hydrogen bonds and salt bridges of the C-terminus (rainbow) with residues of the N-terminal domain (grey). Distances and involved residues are indicated.

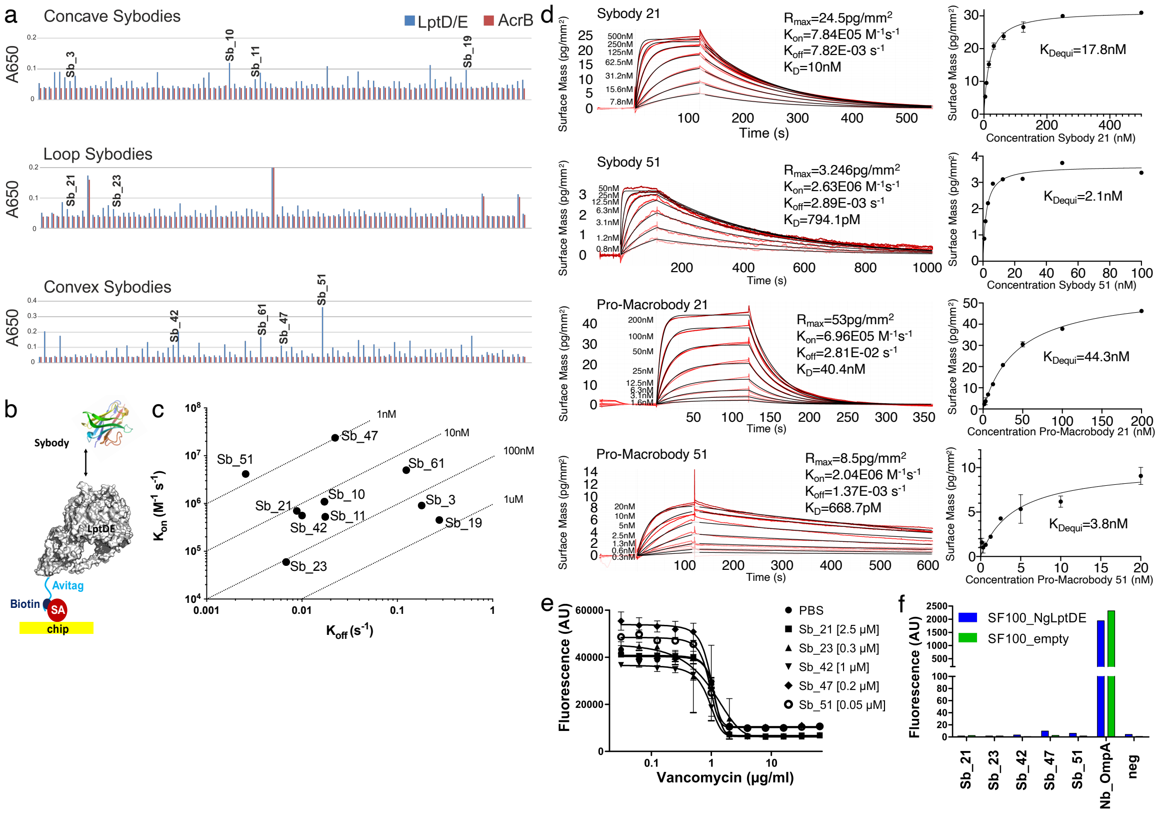

Supplementary Fig. 7: Sybody generation, characterization and comparison to PMb versions. (a) ELISA analysis of sybodies from the concave (top panel), loop (middle panel) and convex (lower panel) library. ELISA signals towards NgLptDE are shown in blue and control ELISA signals against AcrB are shown in red. Sybodies that were sequenced, purified and analysed by waveguide interferometry are indicated by providing the respective Sb_# numbers above the bars. (b) Illustration of the selection procedure using immobilized NgLptDE on Streptavidin chips as used for waveguide interferometry. (c) Evaluation of the binding kinetics of NgLptDE-positive sybodies using waveguide interferometry. (d) Binding kinetics of Sb21 and Sb51 (top panels) and PMb21/51 lower panels. KD values for Sb21, Sb51, PMb21 and PMb51 are 10 nM, 794.1 pM, 40.4 nM and 668.7 pM respectively. For each measurement, n=2 independent experiments and the error bars indicate the SD of the mean. (e) Inhibition assay of *N. gonorrhoeae* growth in the presence of sybodies at concentrations indicated in the legend and varying concentrations of vancomycin. For each measurement, n=3 independent experiments and the error bars indicate the SD of the mean. (f) Binding of fluorescently labeled sybodies on the surface of *E. coli* SF100 cells expressing NgLptDE (blue bars) or the same cells containing an empty vector control (green bars). Anti-OmpA Nbs are used as positive control (Nb_OmpA). No binders were added to the negative control (neg). Source data are provided as a Source Data file.

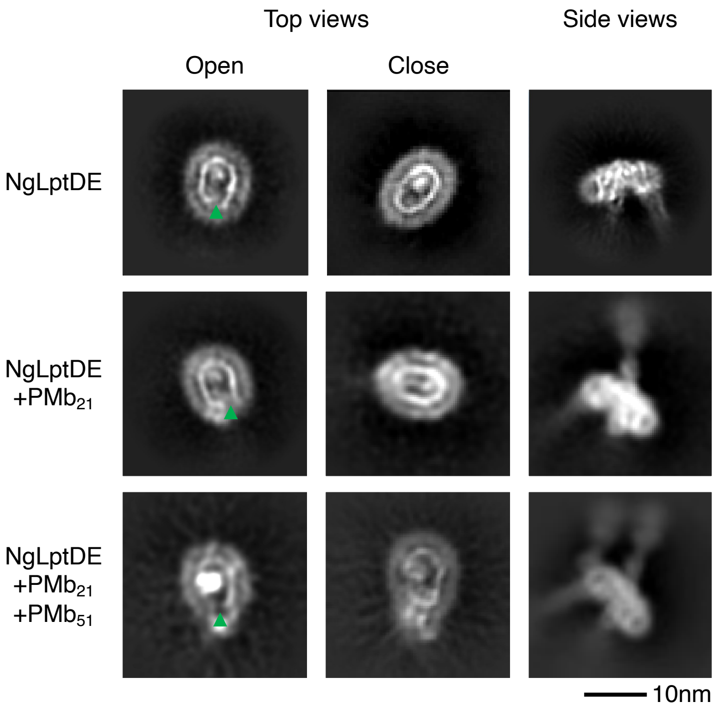

Supplementary Fig. 8: Open and closed conformation for various NgLptDE complexes. Comparison of closed and open 2D class averages obtained for NgLptDE in absence or presence of PMbs. The green arrow head highlights the absence of density at the luminal gate observed for the open conformation of the LptD β-barrel.

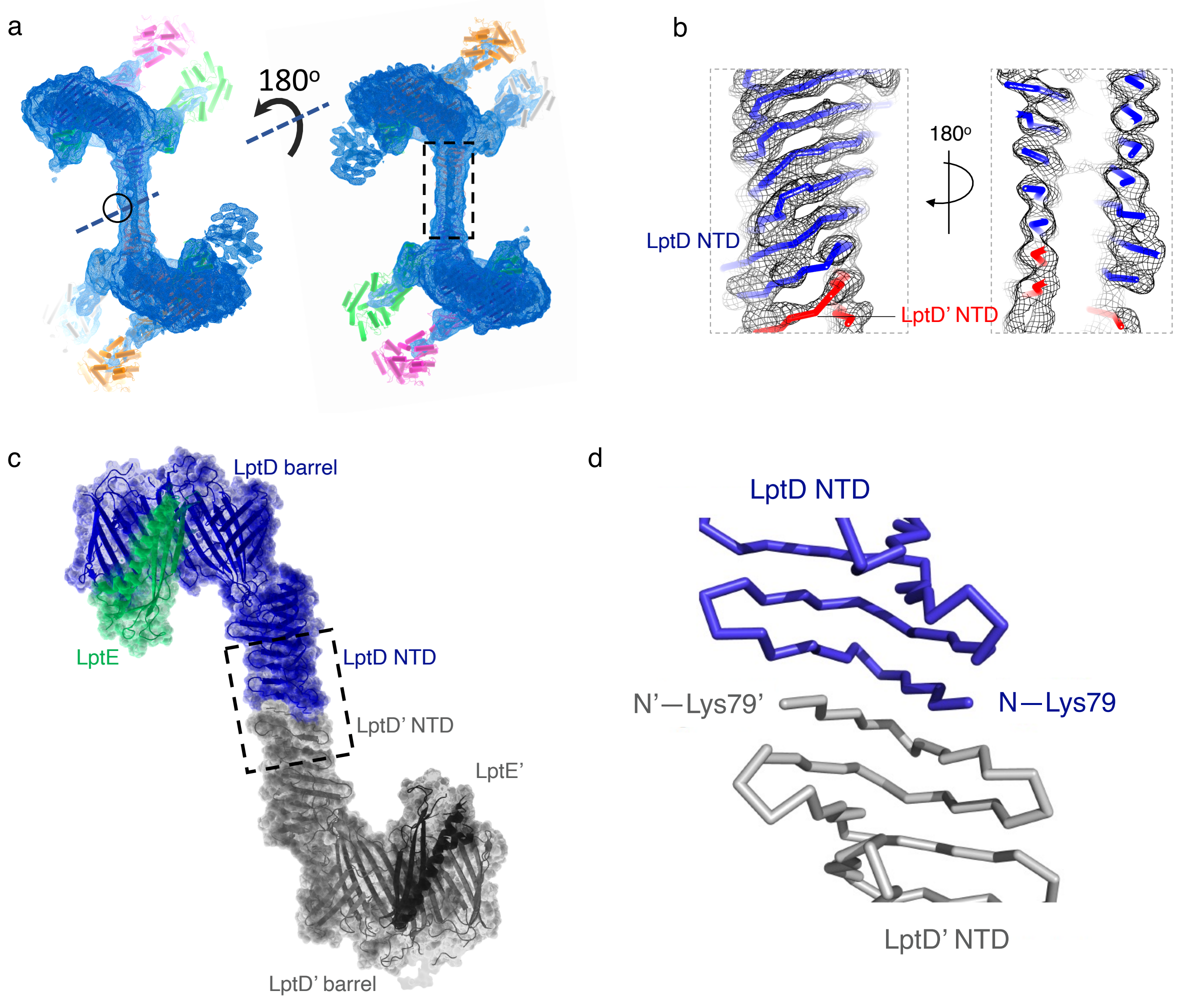

Supplementary Fig. 9: Head-to-head dimers of NgLptDE-PMb$_{21/51}$ complexes. (a) Map of the head-to-head dimer of the NgLptDE-PMb$_{21/51}$ complex. (b) Enlarged view of the stalk formed by the N-terminal regions boxed in (a). (c) Model of the dimer with enlarged view of the head-to-head dimerization by β-strand complementation shown in (d).

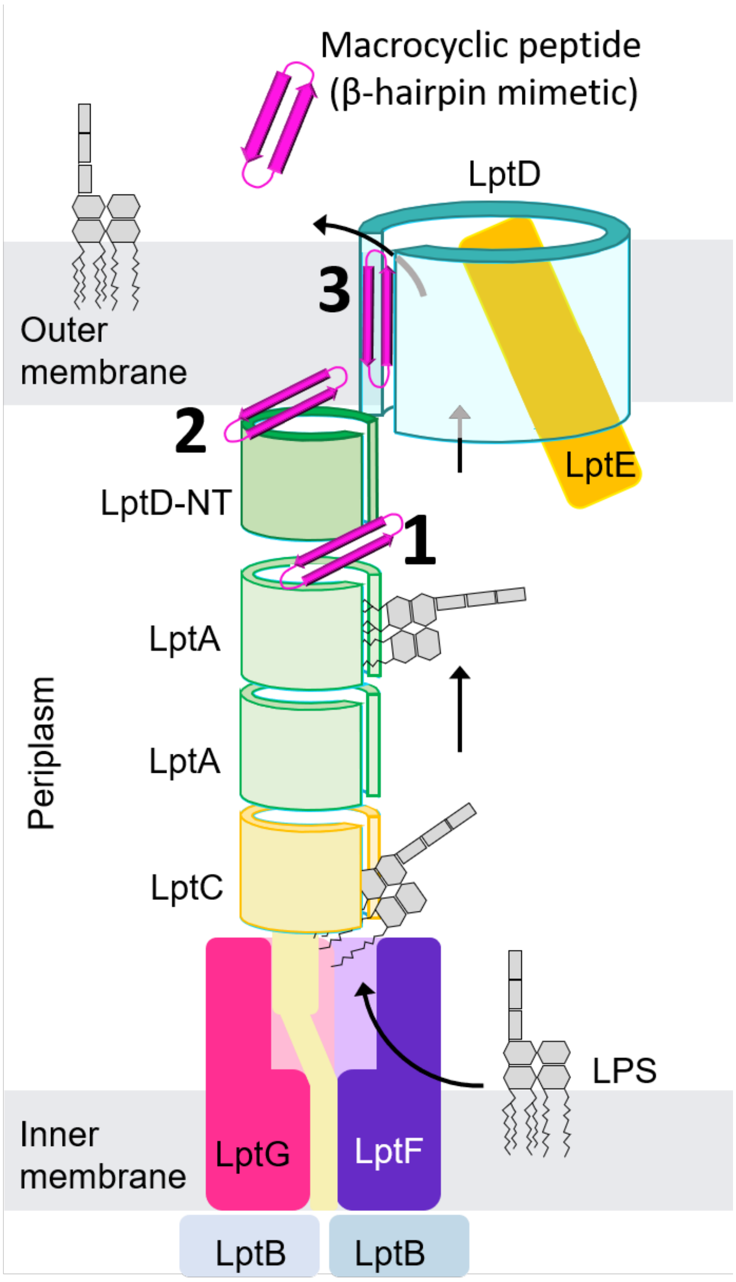

Supplementary Fig. 10: Binding sites for peptidomimetics targeting LptD. LPS is transported through the trans-envelope complex via the Lpt-pathway (black arrows). Blocking of this route by peptidomimetics that bind to terminal β-strands is a promising strategy to interfere with OM biogenesis of gram-negative pathogenic bacteria. Thanatin, a naturally occurring peptide, binds to the N-terminus of LptD next to described LptA interactions and disrupts the assembly of the LPS-route (1). Attractive binding sites for peptidomimetics are also present at the end of the N-terminal domain of LptD (2) like the β-augmentation by the CDR3 loop of PMb51 we observed in the structure and in particular in the cleft between β1 and β26 of a transiently open LptD barrel (3). The latter would be most accessible for targeting compounds as the OM would not need to be traversed.

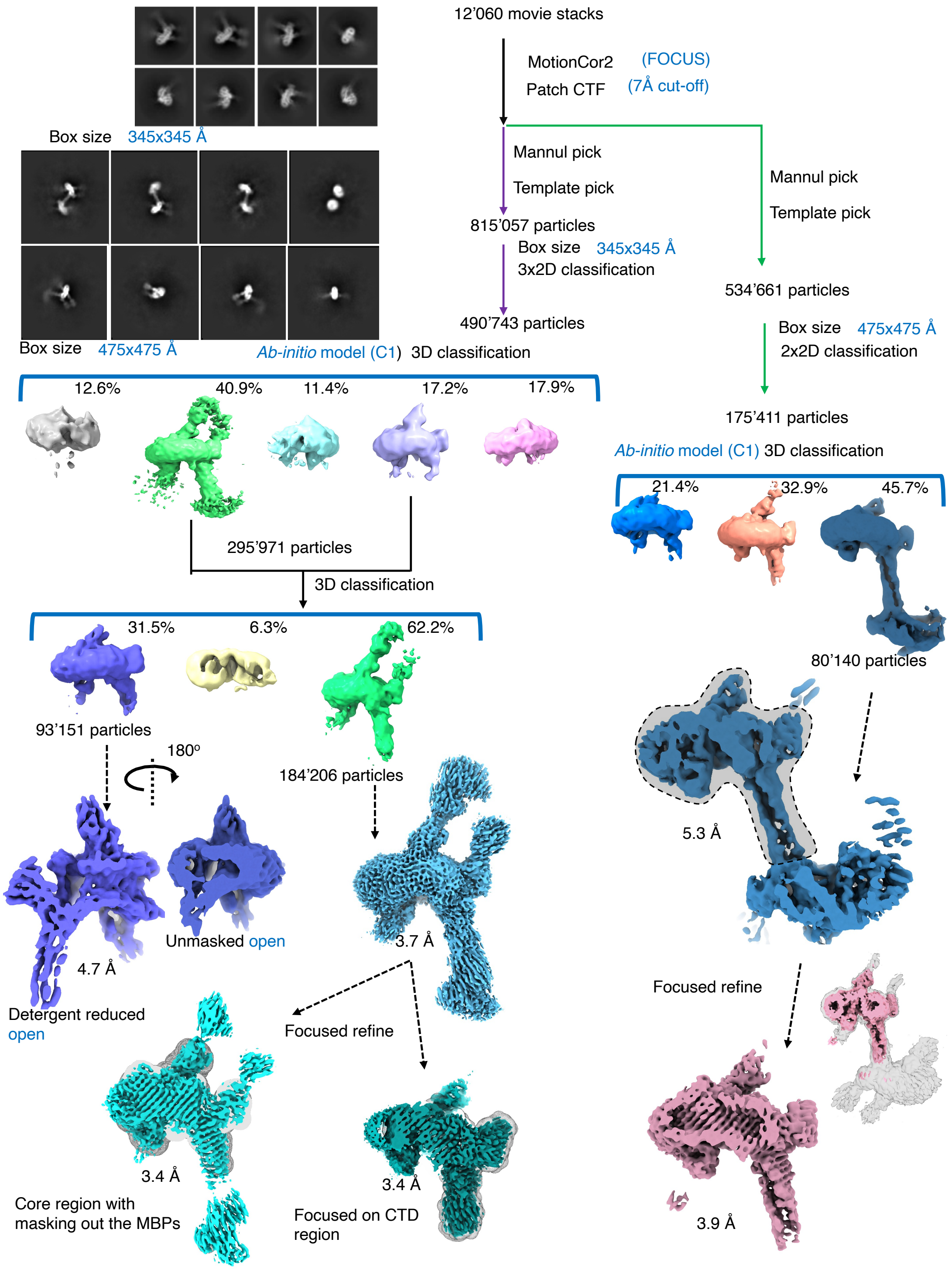

108

109 Supplementary Fig. 11: Processing workflow for the NgLptDE-PMb$_{21}$-PMb$_{51}$ complex. The mask
110 used during refinement are shown in light gray.

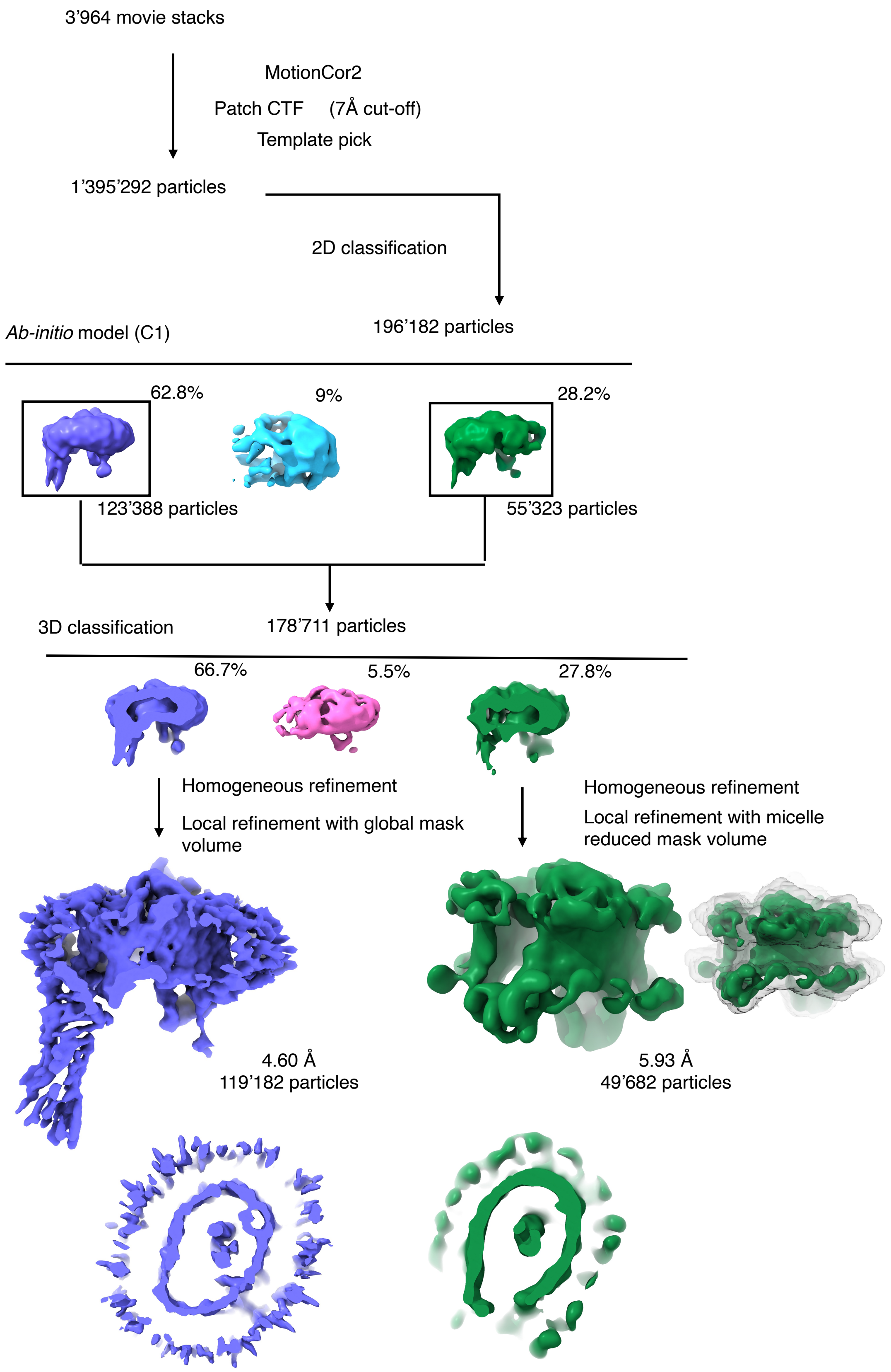

111

112 Supplementary Fig. 12: Processing workflow for the NgLptDE complex. The mask used during
113 refinement are shown in light gray. A section of the LptD barrel is represented for both the closed and
114 open conformation.

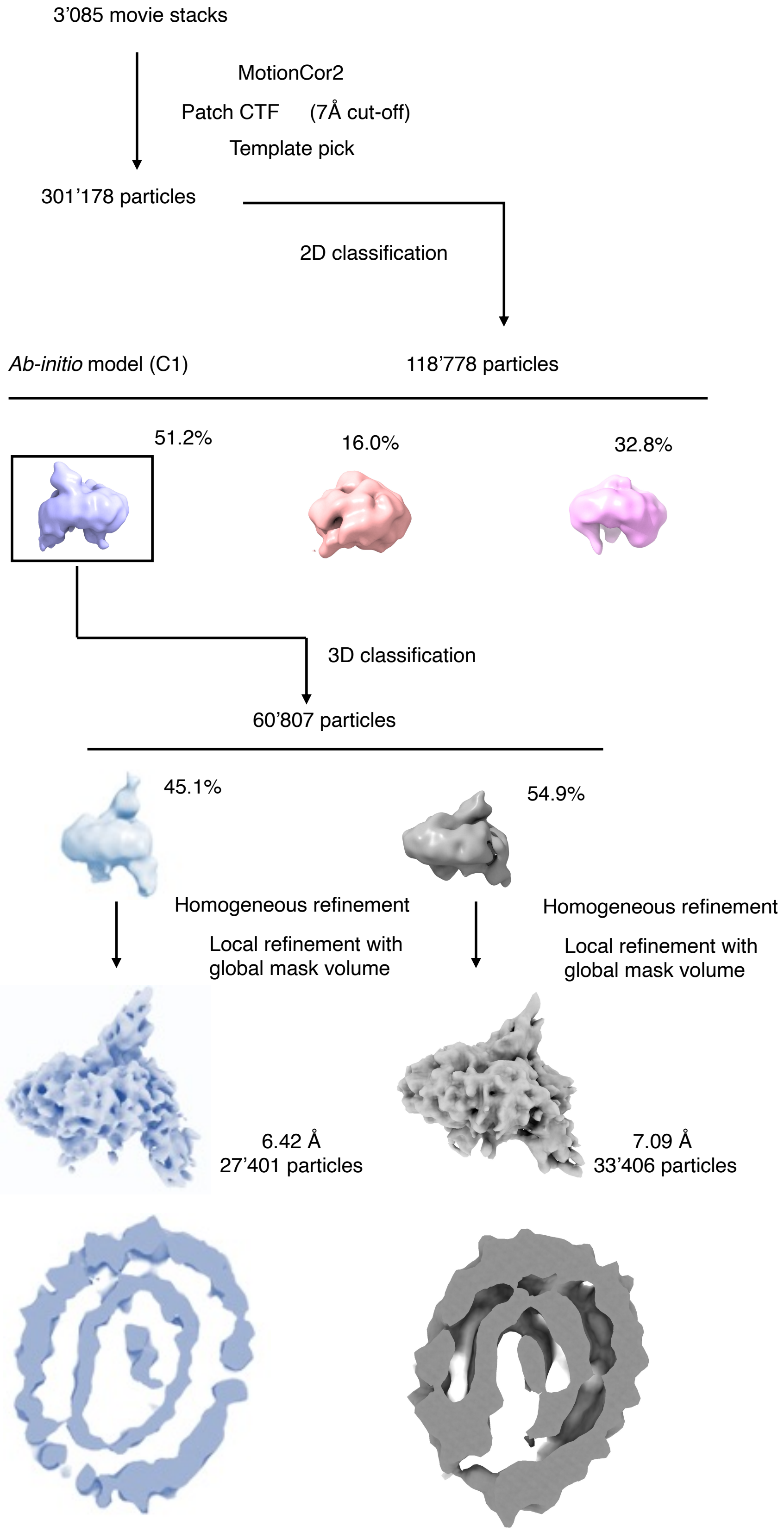

115

116 Supplementary Fig.13: Processing workflow for the NgLptDE-PMb$_{21}$ complex. A section of the LptD
117 barrel is represented for both the closed and open conformation.

	NgLptDE-PMb$_{21}$-PMb$_{51}$					Apo NgLptDE	
						Monomeric fraction	Dimeric fraction
Microscope	FEI Titan Krios					FEI Titan Krios	FEI Titan Krios
Voltage (keV)	300					300	300
Camera	Gatan K2-Summit					Gatan K2-Summit	Gatan K2-Summit
Electron exposure (e-/Å^2)	1.42					1.33	1.33
Energy filter slit width (eV)	20 (Gatan Quantum-LS (GIF))					20 (Gatan Quantum-LS (GIF))	20 (Gatan Quantum-LS (GIF))
Pixel size (Å)	0.82					0.64	0.64
Defocus range (µm)	(−0.8) - (−2.8)					(−0.8) - (−2.8)	(−0.8) - (−2.8)
Magnification (nominal)	60’975x (165kx)					78’125x (215kx)	78’125x (215kx)
Number of frames per movie	35					45	45
Number of good micrographs	12’060					3’964	1’578
Refinement procedure	As monomer			As dimer		As monomer	As dimer
	Overall	Focused on C-terminal	Open conformation	Overall	Focused on dimerization interface		
Initial particles (Before/After 2D cleaning)	815’057/490’743			534’661/175’411		1’395’292/196’182	112’358/32’079
Final particles	184’206	184’206 (softmask)	93’151	80’140	80’140 (softmask)	119’115	32’079
Map resolution (Å)	3.40	3.43	4.72	5.27	3.93	4.60	7.46
FSC threshold	0.143	0.143	0.143	0.143	0.143	0.143	0.143
Map resolution range (Å)	20 - 2.60	20 - 2.90	20 - 4.10	20 – 4.30	20 – 3.40	20 - 4.0	20 – 6.50

Supplementary Table 1: Cryo-EM data collection, refinement and statistics.

	**PMb_{21}**
**Data collection**	
Number of crystals	1
Space group	I2
Cell dimensions	
a, b, c (Å)	121.76 87.62 51.90
α, β, γ (°)	90.00 105.82 90.00
No. of reflections	248315
No. of unique reflections	34750
Resolution (Å)	44.25-2.00 (2.05-2.00)*
R_{merge}	0.057 (1.167)
R_{pim}	0.023 (0.469)
$CC_{1/2}$	0.999 (0.722)
$I/\sigma I$	15.1 (1.5)
Completeness (%)	98.1 (96.7)
Redundancy	7.1 (7.0)
**Refinement**	
Resolution (Å)	2.0
No. of reflections	34737
R_{work}/R_{free}	0.193/0.239
No. atoms	
Protein	3766
Ligand	23
Water	153
Other	25
B-factors	
Protein	62.6
Ligand	47.2
Water	54.3
Other	63.4
R.m.s deviations	
Bond length (Å)	0.010
Bond angle (°)	1.021
Ramachadran plot statistics (%)*	
Favoured regions	97.51
Allowed regions	2.29
Disallowed regions	0.21

Supplementary Table 2: Summary of data collection and refinement statistic for the PMb21 structure obtained by X-ray crystallography. (*) numbers in brackets denote highest resolution shell.
