# Supplementary material for: Cryo-EM structures of a LptDE transporter in complex with Pro-macrobodies offer insight into lipopolysaccharide translocation
Source: Nat Commun. 2022 Apr 5;13:1826. doi: 10.1038/s41467-022-29459-2 (PMC8983717; doi:10.1038/s41467-022-29459-2)
Supplement: Supplementary file 2 — Reporting Summary [file 41467_2022_29459_MOESM2_ESM.pdf]

nature portfolio

Corresponding author(s): Michael Hennig

Last updated by author(s): Feb 24, 2022

Reporting Summary

Nature Portfolio wishes to improve the reproducibility of the work that we publish. This form provides structure for consistency and transparency in reporting. For further information on Nature Portfolio policies, see our Editorial Policies and the Editorial Policy Checklist.

Statistics

For all statistical analyses, confirm that the following items are present in the figure legend, table legend, main text, or Methods section.

n/a	Confirmed	
☐	☒	The exact sample size (*n*) for each experimental group/condition, given as a discrete number and unit of measurement
☐	☒	A statement on whether measurements were taken from distinct samples or whether the same sample was measured repeatedly
☒	☐	The statistical test(s) used AND whether they are one- or two-sided<br>*Only common tests should be described solely by name; describe more complex techniques in the Methods section.*
☒	☐	A description of all covariates tested
☒	☐	A description of any assumptions or corrections, such as tests of normality and adjustment for multiple comparisons
☐	☒	A full description of the statistical parameters including central tendency (e.g. means) or other basic estimates (e.g. regression coefficient) AND variation (e.g. standard deviation) or associated estimates of uncertainty (e.g. confidence intervals)
☒	☐	For null hypothesis testing, the test statistic (e.g. *F*, *t*, *r*) with confidence intervals, effect sizes, degrees of freedom and *P* value noted<br>*Give P values as exact values whenever suitable.*
☒	☐	For Bayesian analysis, information on the choice of priors and Markov chain Monte Carlo settings
☒	☐	For hierarchical and complex designs, identification of the appropriate level for tests and full reporting of outcomes
☒	☐	Estimates of effect sizes (e.g. Cohen's *d*, Pearson's *r*), indicating how they were calculated

Our web collection on statistics for biologists contains articles on many of the points above.

Software and code

Policy information about availability of computer code

Data collection	SerialEM version3-6-22, Agilent ChemStation 2.15.26, Unicorn V7.1
Data analysis	Focus v1.1.0, MotionCor2 v1, CTFFIND v4.1, CryoSPARC v2.03, Coot v0.8.9.1, Phenix 1.17-3644, USCF Chimera v1.14, USCF ChimeraX v0.9, Pymol v2.2.3

For manuscripts utilizing custom algorithms or software that are central to the research but not yet described in published literature, software must be made available to editors and reviewers. We strongly encourage code deposition in a community repository (e.g. GitHub). See the Nature Portfolio guidelines for submitting code & software for further information.

Data

Policy information about availability of data

All manuscripts must include a data availability statement. This statement should provide the following information, where applicable:

- Accession codes, unique identifiers, or web links for publicly available datasets
- A description of any restrictions on data availability
- For clinical datasets or third party data, please ensure that the statement adheres to our policy

The EM Map for NgLpTDE (partially open) bound to Pmb21 and PMb51 was deposited in the EMDB under accession code EMD-12990 [https://www.ebi.ac.uk/pdbe/entry/emdb/EMD-12990]. Coordinates for NgLpTDE (partially open) bound to Pmb21 and PMb51 were deposited at the Protein Databank under accession code PDB 7OMM [https://doi.org/10.2210/pdb7OMM/pdb]. Coordinates for the X-Ray structure of PMb21 were deposited at the Protein Databank under accession code PDB 7OMT [https://doi.org/10.2210/pdb7OMT/pdb]. Source data for chromatograms from chromatography runs, grow curves and affinity measurements has been provided as a .xlsx Source Data file (NCOMMS-21-22078A_Source_Data). All other data is available from the authors upon reasonable request (michael.hennig@leadxpro.com)

Field-specific reporting

Please select the one below that is the best fit for your research. If you are not sure, read the appropriate sections before making your selection.

☒ Life sciences ☐ Behavioural & social sciences ☐ Ecological, evolutionary & environmental sciences

For a reference copy of the document with all sections, see nature.com/documents/nr-reporting-summary-flat.pdf

Life sciences study design

All studies must disclose on these points even when the disclosure is negative.

Sample size	Sample size for growth curves and sybody binding to cells was not predetermined. Experiments were repeated twice with similar outcome. Error bars represent the SD of the mean from two experiments. Waveguide interferometry measurements for binding kinetics were repeated twice and error bars denote the SD of the mean. Chromatography runs were performed without applying statistics. Similar outcomes were observed in repeated runs. Example chromatograms are shown in the figures. 12060 micrographs were taken for the NgLptDE-PMB21/PMb51 complex from which 184206 particles were used for the generation of the map of the partially opened state and 93151 particles for the fully open state. A single crystal was used for collecting diffraction data for the structure of PMb21.
Data exclusions	No data was excluded except for micrographs of low quality based on the resolution of the CTF fit as detailed in the Methods section and in Supplementary Table 2.
Replication	Growth inhibition and sybody cell binding assays were repeated twice. Protein purifications were repeatedly performed with similar outcomes. Replication attempts were successful.
Randomization	Samples were not randomized.
Blinding	None of the data was taken blindly or double blind. Blinding is not applicable to this type of study. Particle classification and resolution estimation were performed automatically from processing software packages.

Reporting for specific materials, systems and methods

We require information from authors about some types of materials, experimental systems and methods used in many studies. Here, indicate whether each material, system or method listed is relevant to your study. If you are not sure if a list item applies to your research, read the appropriate section before selecting a response.

Materials & experimental systems

n/a	Involved in the study	
☒	☐	Antibodies
☒	☐	Eukaryotic cell lines
☒	☐	Palaeontology and archaeology
☒	☐	Animals and other organisms
☒	☐	Human research participants
☒	☐	Clinical data
☒	☐	Dual use research of concern

Methods

n/a	Involved in the study	
☒	☐	ChIP-seq
☒	☐	Flow cytometry
☒	☐	MRI-based neuroimaging
